# Multisensory pathogen detection drives rapid escape and shapes host–microbe interactions in *Drosophila* larvae

**DOI:** 10.1371/journal.ppat.1014506

**Published:** 2026-08-12

**Authors:** Olivier Zugasti, Julien Royet

**Affiliations:** Institut de Biologie du Développement de Marseille, Aix-Marseille Université, CNRS UMR, Marseille, France; Cornell University, UNITED STATES OF AMERICA

## Abstract

Larvae of many insects develop immersed in decomposing substrates densely populated with microbes, yet how they detect and respond to pathogenic threats during early life remains poorly understood. Here, we show that *Drosophila melanogaster* larvae exhibit a previously unrecognized rapid escape behavior triggered by food contaminated with metabolically active *Erwinia carotovora carotovora 15* (*Ecc15*), a natural bacterial pathogen of flies and plants. Stationary-phase cells fail to elicit avoidance, demonstrating that larval detection of *Ecc15* depends on bacterial metabolic activity rather than on its mere presence. Using targeted genetic manipulations, we identify two chemosensory pathways required for this response: a gustatory input mediated by the aversion receptor Gr33a and an olfactory input involving the Or49a–Orco complex. Disrupting either pathway abolishes escape, revealing that larvae rely on coordinated gustatory and olfactory signals to evaluate and respond to microbial dangers. Functionally, escape limits contact time with contaminated substrates and enables larvae to reach uncontaminated food, partially mitigating the developmental impact of early pathogen exposure. However, dispersing larvae also transfer viable bacteria to new substrates, indicating that this avoidance behavior concurrently promotes pathogen spread. Together, these findings establish the first example of a rapid, multisensory escape behavior induced by a natural pathogen in *Drosophila* larvae and provide a tractable model for dissecting how microbial cues guide behavioral decision-making and influence pathogen dissemination.

## Author summary

Animals often encounter microbes while feeding, yet the mechanisms by which they detect and respond to harmful bacteria remain poorly understood. Here, we show that *Drosophila melanogaster* larvae rapidly abandon food contaminated with *Erwinia carotovora carotovora 15* (*Ecc15*), a natural bacterial pathogen. Larvae initially approach contaminated food but retreat after contact. This escape behavior requires live, metabolically active bacteria and depends on both gustatory and olfactory inputs. Specifically, larvae use a gustatory receptor that detects aversive contact cues together with an olfactory receptor that senses bacterial odors. Blocking either pathway abolishes escape. Although larvae can relocate to uncontaminated food when alternative resources are available, they also transfer viable bacteria to new substrates. Our findings reveal a previously unknown multisensory avoidance response in insect larvae and highlight how host behavioral responses can simultaneously reduce exposure to contaminated substrates and facilitate pathogen dissemination.

## Introduction

Animals developing in microbe-rich environments must continuously balance the need to acquire nutrients with the risk of encountering pathogenic microorganisms [1,2]. In holometabolous insects such as *Drosophila melanogaster*, this constraint is particularly pronounced during the larval stage, when individuals feed almost continuously within decaying fruits and fermenting substrates densely populated with bacteria and yeasts [3,4]. This ecology has shaped a robust and well-characterized innate immune system in both larvae and adults, enabling the detection and control of diverse microbial threats [5]. Several natural bacterial pathogens of *Drosophila*, including *Erwinia carotovora carotovora 15* (*Ecc15* now reclassified as *Pectobacterium carotovorum*), *Pseudomonas entomophila*, and *Serratia marcescens*, have therefore become powerful models for dissecting host–microbe interactions and infection-induced physiological alterations [6–8].

Among these microbes, *Ecc15* is a Gram-negative phytopathogen of the *Enterobacteriaceae* family that proliferates in damaged plant tissues and causes soft-rot disease [9]. Its ecological niche overlaps with that of *Drosophila*, and adult flies can naturally acquire and disseminate *Erwinia* between plant hosts [10–12]. Ingestion of *Ecc15* triggers strong immune activation, including systemic immune deficiency pathway (IMD) stimulation through detection of diaminopimelate-type peptidoglycan (PGN) [6,13–15] and, in adults, uracil-dependent DUOX activation in the gut epithelium, leading to the production of reactive oxygen species [16]. *Ecc15* also expresses the virulence factor Evf (*Erwinia* virulence factor), which promotes bacterial persistence in the larval gut and modifies host physiology, including delaying gut clearance and altering feeding behavior [17,18]. These features indicate that *Drosophila* naturally encounter *Ecc15* and raise the possibility that host strategies may contribute to limiting bacterial contact.

Across animal taxa, behavioral responses such as avoidance, withdrawal, and refuge seeking can occur rapidly upon exposure to contaminated substrates and shape subsequent host–microbe interactions [19]. In adult *Drosophila*, several dedicated sensory pathways mediate avoidance of harmful microbes or their metabolites, including olfactory detection of fungal geosmin and phenolic volatiles through the odorant receptors Or56a [20] and Or46a [21], as well as gustatory detection of bacterial lipopolysaccharides (LPS) through the transient receptor potential cation channel A1 (TRPA1) expressed in Gr66a-positive bitter sensory neurons [22]. These findings demonstrate that adults rely on both olfaction and gustation to detect and respond to microbial cues.

In contrast, much less is known about corresponding strategies in larvae, whose sensory architecture, foraging habits, and ecological constraints differ substantially [23–25]. This gap is notable given that larvae develop immersed in their food substrate, ingesting large quantities of microbes while relying primarily on close-range chemical cues to assess food quality. One previous study showed that larvae can abandon food contaminated with *P. entomophila*, indicating that pathogen-associated avoidance behaviors can occur during the larval stage [26]. However, the sensory mechanisms underlying these responses, the extent to which they can support rapid escape from contaminated substrates, and their broader ecological implications remain poorly understood. In particular, the sensory and neuronal mechanisms involved in detecting pathogen-associated cues, as well as the consequences of these behaviors for host exposure and pathogen dissemination, have not been explored. Here, we address these questions by analyzing larval behavioral responses to food contaminated with *Ecc15* and by investigating the sensory pathways underlying escape behavior and its ecological outcomes.

## Results

### Metabolically active *Ecc15* triggers dispersal behavior in *Drosophila* larvae

**Fig 1:**
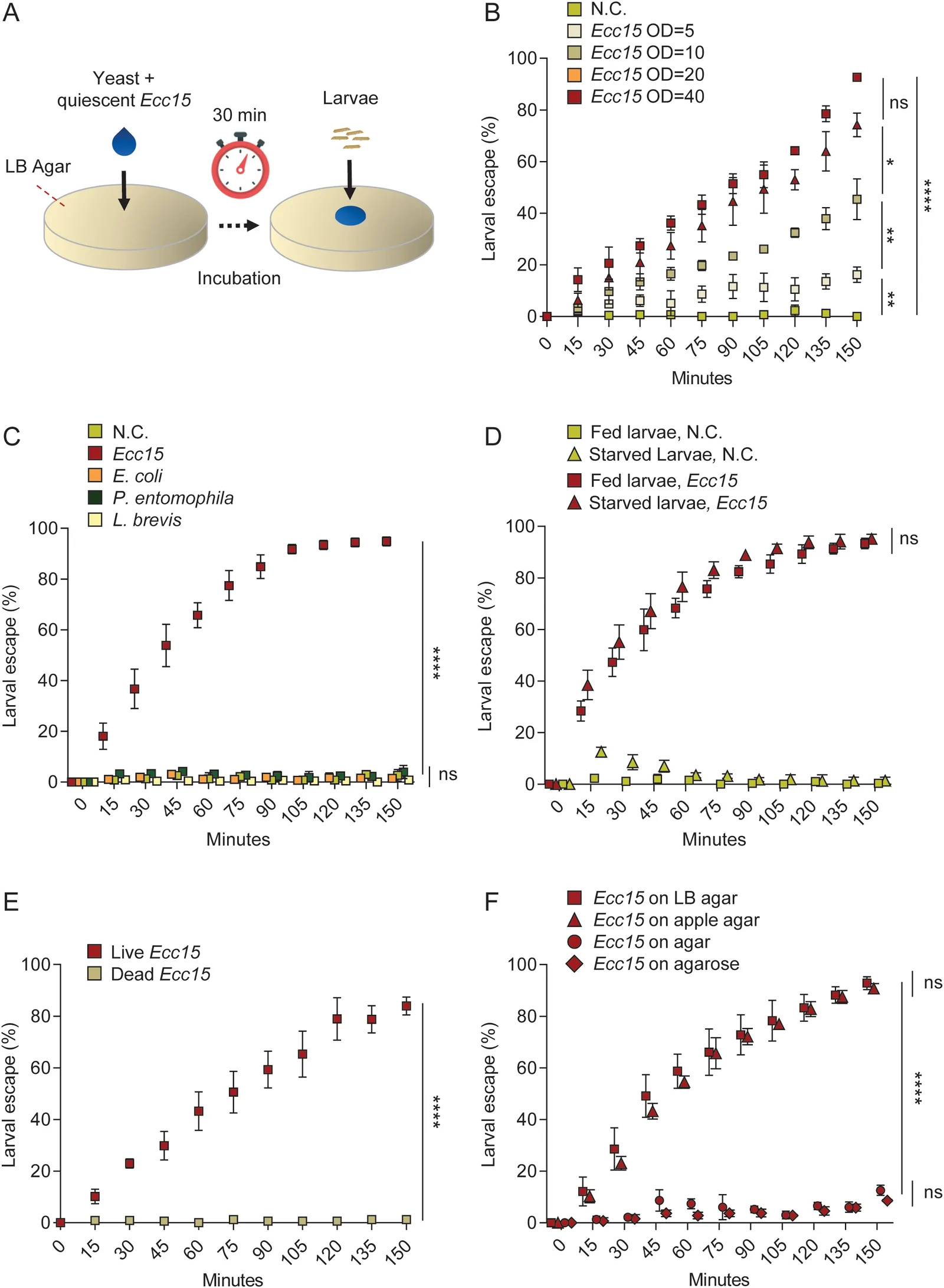
Metabolically active *Ecc15* cells trigger rapid and dose-dependent escape of *Drosophila* larvae. **(A)** Schematic of the assay used to evaluate larval responses to *Ecc15*-contaminated food. **(B)** Quantification of larval dispersal from uncontaminated yeast (N.C.) and yeast contaminated with increasing concentrations of *Ecc15* (OD_600_ = 5, 10, 20, and 40) over time. **(C)** Comparison of larval dispersal on uncontaminated yeast versus yeast contaminated with *Ecc15*, *E. coli, P. entomophila* and *L. brevis* at identical bacterial densities (OD_600_ 40). **(D)** Escape behavior of fed larvae and larvae starved for 5 h on uncontaminated or *Ecc15*-contaminated yeast. **(E)** Dispersal of larvae exposed to live versus heat-killed *Ecc15*. **(F)** Effect of nutrient-rich LB agar, carbohydrate-rich but nitrogen-limited apple agar, low-nutrient agar, and nutrient-free agarose substrates on *Ecc15*-induced escape. For this and all subsequent figures, quantified dispersal assay results are shown as mean ± SD of technical triplicates from a representative biological experiment. Each experiment was independently repeated at least three times, using groups of ≥40 larvae per replicate. Statistical analyses were performed using a mixed-effects model (REML) with time and condition as fixed factors, (ns = not significant; *p < 0.05; **p < 0.01; ****p < 0.0001). Comparisons between selected conditions are shown. For all experiments, raw data and full statistical outputs are provided in S1 Data.

To characterize larval behavior when exposed to a source of food contaminated with *Ecc15*, baker’s yeast (*Saccharomyces cerevisiae*), a nutritive substrate supporting optimal larval growth [27], was mixed with increasing concentrations of stationary-phase *Ecc15* cultured overnight (S1A Fig). This yeast–bacteria mixture was then placed on nutrient-rich LB agar plates and left to incubate at room temperature for 30 minutes, a duration corresponding to the early phase of bacterial regrowth in static liquid culture (S1B Fig). Wild-type (*w¹¹¹⁸*) second-instar larvae were subsequently transferred directly onto the contaminated food for behavioral assays (Fig 1A).

Exposure to *Ecc15*-contaminated food caused larvae to disperse within minutes, and this response increased both over time and with bacterial load (Fig 1B; corresponding CFU values in S1C Fig). At bacterial densities equivalent to OD_600_ 20–40, more than 80% of the larvae had dispersed within 2.5 hours, whereas lower concentrations (OD_600_ 5–10) elicited a weaker initial response. Importantly, CFU counts remained stable during the 30 min pre-incubation period on LB agar across all tested concentrations, indicating that differences in larval behavior are unlikely to result from differential bacterial growth prior to larval exposure (S1D Fig). Once larvae moved away from the contaminated food, they remained off the substrate for the rest of the observation period, suggesting active avoidance (Figs 1B, S2). After 24 hours, nearly all larvae had abandoned the *Ecc15*-contaminated yeast at every concentration tested, whereas larvae consistently remained on uncontaminated food (S2 Fig). Based on the rapidity and reproducibility of the response at OD_600_ 40, this concentration was used in subsequent experiments unless otherwise indicated.

The robustness of this dispersal behavior was verified across multiple *Drosophila melanogaster* genetic backgrounds including *Canton-S*, *Oregon-R*, and *yellow white* larvae, all of which displayed rapid escape from *Ecc15*-contaminated food (S3 Fig). To examine the temporal stability of this behavior, yeast–*Ecc15* mixtures stored on LB agar plates at room temperature for 2, 4, or 6 days were tested. In every case, larvae continued to disperse robustly and extensively, demonstrating that this escape response is stable and independent of the age of the bacterial preparation (S4 Fig).

To evaluate the specificity of the escape response, larvae were exposed to yeast mixed with different bacterial species, including the facultative anaerobic *Escherichia coli (E. coli)*, a non-pathogenic member of the *Enterobacteriaceae* family, *Pseudomonas entomophila (P. entomophila)*, an established entomopathogen [7], and *Levilactobacillus brevis (L. brevis)*, a common member of the *Drosophila* microbiota generally considered a commensal, but which can exert deleterious effects depending on host immune status and microbiota composition [16,28]. All bacterial suspensions were prepared from stationary-phase cultures and applied to LB agar plates using the same procedure as for *Ecc15.* While larvae rapidly abandoned food contaminated with *Ecc15*, they remained on yeast containing *E. coli*, *P. entomophila*, *L. brevis*, as well as on uncontaminated yeast (Figs 1C, S5 Fig and S1–S3 Movies). These results show that the dispersal behavior is not a general response to bacterial presence or pathogenicity, but is instead likely triggered by cues specific to *Ecc15*.

Since starvation has been shown to influence pathogen-induced behavioral decisions in larvae exposed to *P. entomophila* [26], the impact of larval nutritional state on escape from *Ecc15*-contaminated food was examined. No significant difference in dispersal was observed between fed larvae and larvae starved for 5 h on PBS-soaked filter paper. Both groups rapidly abandoned *Ecc15*-contaminated food, indicating that this escape response is largely insensitive to larval nutritional state (Fig 1D).

To determine whether bacterial viability is required for larval dispersal, larvae were exposed to either live or heat-killed *Ecc15*. Only live *Ecc15* bacteria induced dispersal, whereas heat-killed bacteria produced no response (Fig 1E). Larvae also moved away from food containing live *Ecc15* mixed with either metabolically active or inactive yeast, showing that the behavior is driven by bacterial rather than yeast-derived signals (S6 Fig). Consistently, larvae withdrew from *Ecc15*-contaminated ripe mashed banana but remained on uncontaminated food, demonstrating that this behavior occurs across distinct nutritive substrates (S7 Fig). Together, these findings support the conclusion that larval dispersal is triggered specifically by bacterial factors associated with live *Ecc15* and is not restricted to a particular food substrate.

Requirements for bacterial metabolism were further tested by placing yeast–*Ecc15* mixtures on media with varying nutrient compositions, including nutrient-rich LB agar, carbohydrate-rich but nitrogen-limited apple agar, low-nutrient agar, and nutrient-free agarose. Larvae dispersed rapidly on both LB and apple-agar, whereas no dispersal was observed on agar or agarose, where bacterial metabolic activity is expected to be minimal or absent (Figs 1F and S8). These findings indicate that larval escape requires metabolically active bacteria and occurs only under conditions that support bacterial metabolic activity, independent of substrate nutrient composition.

Taken together, these results demonstrate that *Drosophila melanogaster* larvae exhibit a rapid, robust dispersal response to *Ecc15*-contaminated food, triggered by signals from metabolically active bacteria, and dependent on substrate-driven bacterial metabolic activity.

### Larval avoidance of *Ecc15* emerges after initial non-selective food approach

**Fig 2:**
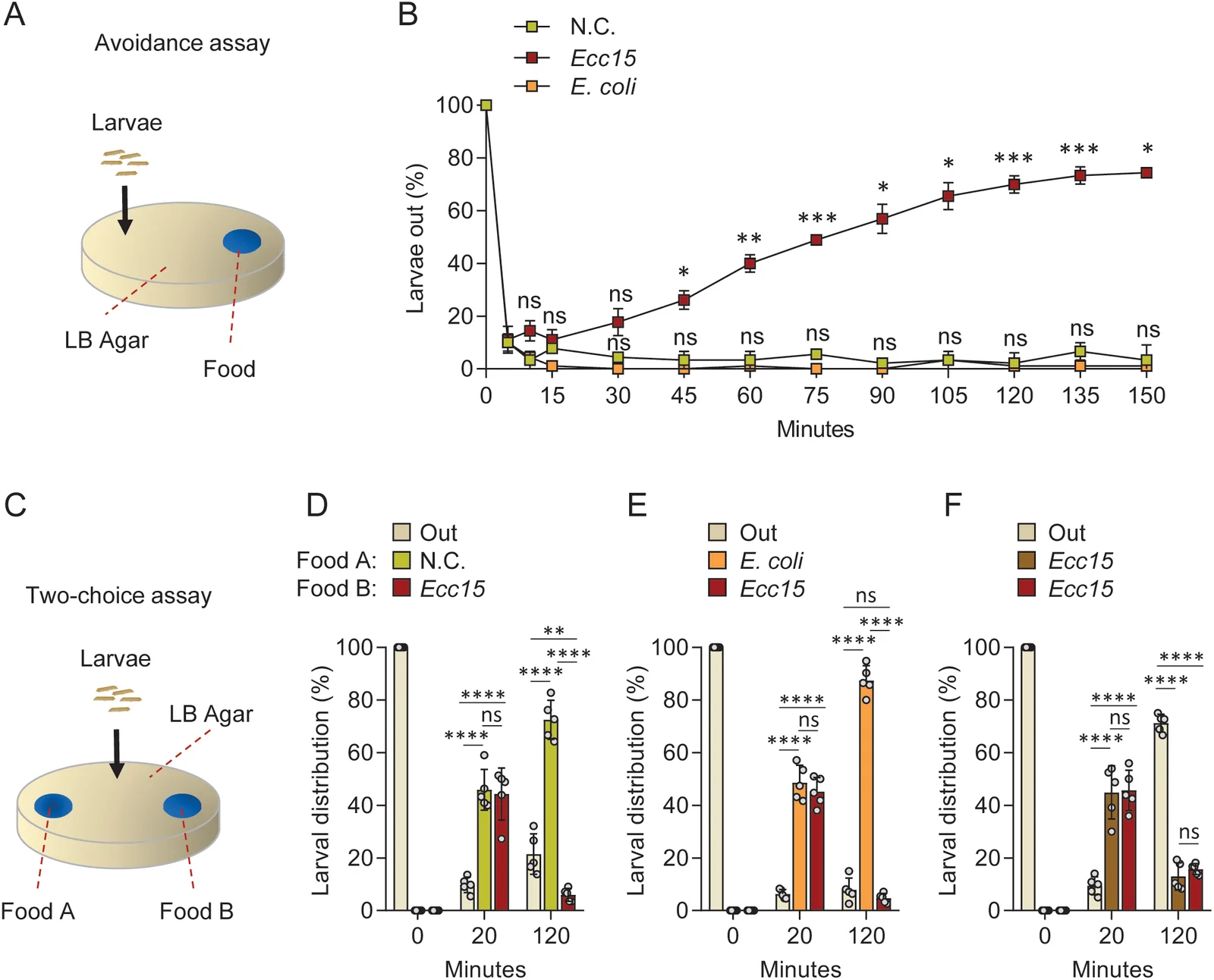
Initial non-selective approach precedes larval escape from *Ecc15*-contaminated food. **(A)** Schematic of the assay monitoring larval behavior toward a distant food source. **(B)** Quantification of larvae located outside the food over time when placed at a distance from uncontaminated yeast (N.C.) or yeast contaminated with *Ecc15.* or *E. coli*. Data are presented as means ± SD of technical triplicates using groups of 30 Larvae. Statistical analyses were performed using a mixed-effects model (REML) with time and condition as fixed factors, followed by Tukey’s post-hoc tests comparing uncontaminated food with either *Ecc15*-contaminated or *E. coli*-contaminated food conditions (ns = not significant; *p < 0.05; **p < 0.01; ***p < 0.001). **(C)** Schematic of the binary food-choice assay. **(D-F)** Distribution of larvae at 0, 20, and 120 min in food-choice assays when presented with paired patches: uncontaminated versus *Ecc15*-contaminated yeast **(D)**; *E. coli* versus *Ecc15*-contaminated yeast **(E)**; two *Ecc15*-contaminated patches **(F)**. Data are presented as means ± SD. Individual points represent experimental replicates (each consisting of groups of ≥ 30 larvae). Statistical analyses were performed using Fisher’s exact test (ns = not significant; **p < 0.01; ****p < 0.0001).

To determine whether *Drosophila* larvae require direct contact with *Ecc15* contaminated yeast to initiate dispersal, an avoidance assay was performed. Larvae were positioned at a distance from the food source under three conditions: uncontaminated yeast, yeast inoculated with *E. coli*, or yeast inoculated with *Ecc15* (Fig 2A). In all conditions, larvae rapidly oriented toward the food and entered it, confirming an attraction to nutritional cues (Fig 2B). However, upon contacting yeast contaminated with *Ecc15*, larvae displayed a rapid escape response and left the substrate shortly thereafter (Fig 2B). By contrast, larvae that reached uncontaminated yeast or yeast containing *E. coli* remained on the food throughout the assay (Fig 2B). These observations indicate that larvae do not detect *Ecc15* at a distance but instead respond to cues perceived upon direct contact with the contaminated food.

To examine larval behavior in the presence of multiple food options, a two-choice assay was conducted. Larvae were placed equidistant between two food patches, and three pairwise comparisons were tested: uncontaminated yeast versus yeast inoculated with *Ecc15*; *E. coli* versus *Ecc15* contaminated yeast; and two patches both containing *Ecc15* (Fig 2C). Larval positions were recorded at 20 and 120 minutes to capture their initial orientation and later decision-making. After 20 minutes, larvae reached one of the food patches in all conditions without showing any preference, indicating that early orientation is not influenced by bacterial contamination (Fig 2D–2F). By 120 minutes, however, clear differences emerged: larvae moved away from *Ecc15*-contaminated food and preferentially occupied uncontaminated or *E. coli*-associated food (Fig 2D–2E). When both patches contained *Ecc15*, larvae abandoned the food entirely and accumulated on the surrounding agar, consistent with a broad avoidance response in the absence of an alternative food source (Fig 2F). These findings demonstrate that *Drosophila* larvae initially approach food without discrimination but retreat upon contact with *Ecc15*-contaminated substrates, subsequently redirecting their movement toward innocuous food sources. Combined with previous evidence that dispersal depends on bacterial metabolic activity rather than mere presence, these results suggest that contact-based detection of *Ecc15* involves specific bacterial metabolites or virulence-associated factors.

### Major *Ecc15*-derived factors or canonical immune and nociceptive signaling do not mediate larval dispersal behavior

**Fig 3:**
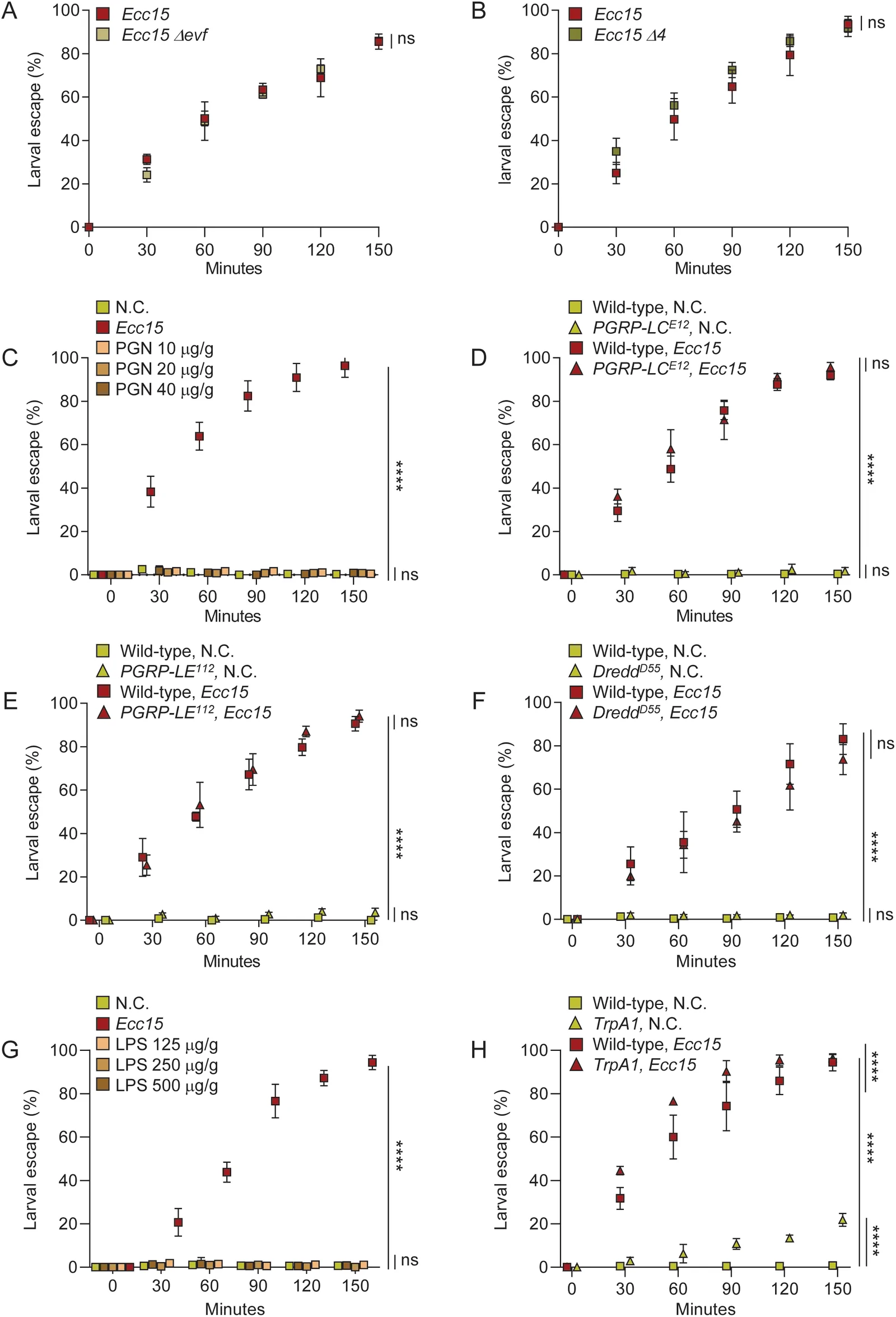
*Ecc15*-induced larval dispersal occurs independently of major bacterial factors, IMD immune signaling, and TRPA1 nociception. **(A)** Larval response to yeast contaminated with wild-type *Ecc15* or the *evf*-deficient mutant (*Ecc15* Δ*evf*). **(B)** Escape behavior induced by wild-type or uracil-deficient *Ecc15* (*Ecc15* Δ*4*). **(C)** Larval dispersal on food supplemented with increasing concentrations of purified PGN (10, 20, 40 μg/mL). **(D–F)** Dispersal of IMD pathway mutants: *PGRP-LC*^*E12*^ **(D)**, *PGRP-LE*^*112*^ **(E)**, and *Dredd*^*D55*^ (F) on uncontaminated yeast and yeast mixed with *Ecc15*. **(G)** Escape response to yeast supplemented with purified LPS (125, 250, 500 µg/mL). **(F)** *TrpA1* mutant larvae behavior on uncontaminated and *Ecc15*-contaminated food. Statistical analyses were performed using a mixed-effects model (REML) with time and condition as fixed factors, (ns = not significant; ****p < 0.0001). Comparisons between selected conditions are shown.

Metabolically active *Ecc15* produces multiple factors, including virulence proteins, metabolites, and structural components, that influence *Drosophila* physiology and potentially cue larval dispersal. The contribution of the secreted virulence factor Evf, which promotes gut colonization and triggers systemic immune activation [17,29], was first assessed. Larvae exposed to yeast contaminated with either wild-type or *evf*-deficient *Ecc15* strains (*Ecc15 Δevf*) [17] dispersed equally, indicating that Evf is not required (Fig 3A).

The role of uracil, a microbe-derived metabolite known to activate DUOX-dependent epithelial defenses in adults [16], was then examined. Comparable levels of dispersal were observed when larvae were exposed to wild-type or uracil-deficient *Ecc15* strains (*Ecc15* Δ*4*) [30], showing that uracil is also dispensable (Fig 3B).

The potential involvement of two conserved Gram-negative cell wall components was next evaluated. Although *Ecc15*-derived PGN is a strong activator of IMD signaling [13,15] and can induce aversive feeding responses in adult flies [31], supplementation of yeast with increasing concentrations of purified PGN did not induce dispersal (Fig 3C). In agreement, larvae lacking the PGN receptors PGRP-LC or PGRP-LE [32,33], or the downstream IMD effector Dredd [34], escaped from *Ecc15*-contaminated food similarly to wild-type larvae (Fig 3D–3F). The same approach was then used to assess the role of LPS, which activates TRPA1-dependent avoidance pathways in adult flies [22]. Purified LPS similarly failed to trigger larval dispersal at any of the concentrations tested (Fig 3G). Consistently, loss of *TrpA1* function did not impair dispersal on *Ecc15*-contaminated yeast (Fig 3H).

Altogether, these findings demonstrate that larval escape behavior from *Ecc15*-contaminated food is not triggered by individual bacterial components or metabolites such as Evf, uracil, PGN, or LPS, nor does it depend on canonical IMD signaling or TRPA1-mediated sensing of noxious cues. Instead, the behavior is likely driven by the detection of *Ecc15*-derived signals through alternative sensory pathways.

### Aversive gustatory receptor Gr33a mediates larval dispersal from *Ecc15*-contaminated food

**Fig 4:**
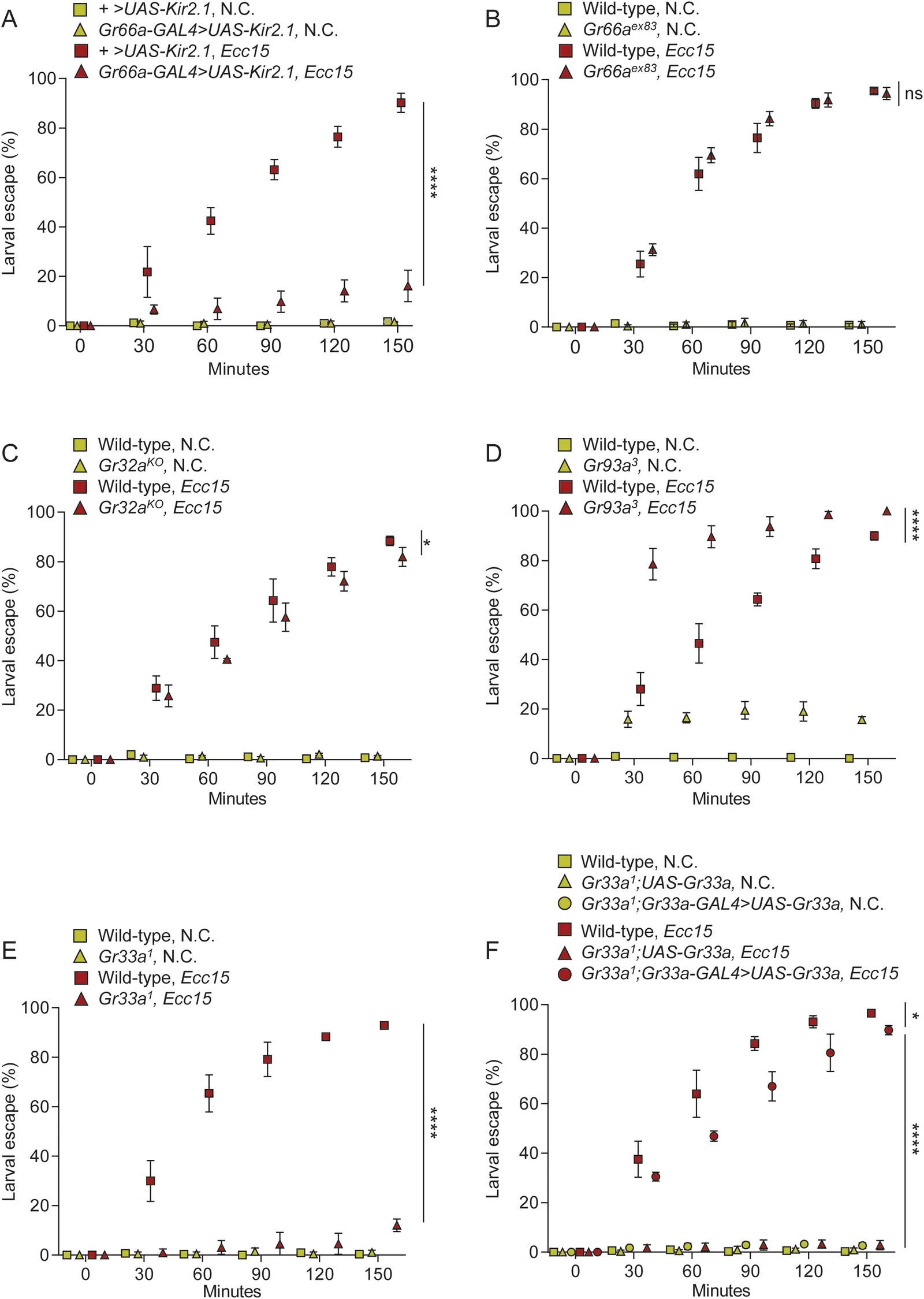
The gustatory receptor Gr33a mediates larval escape from *Ecc15-*contaminated food. **(A)** Larval dispersal following silencing of Gr66a-expressing neurons (*Gr66a-GAL4 > UAS-Kir2.1*) on uncontaminated (N.C.) or *Ecc15*-contaminated food. **(B-E)** Escape response of wild-type larvae and loss-of-function mutants *GR66a*^*ex83*^ **(B)**, *GR32a*^*KO*^ **(C)**, *GR93a*^*3*^ (D) and *Gr33a*^*1*^ (E) exposed to uncontaminated and *Ecc15*-contaminated food. **(F)** Comparison of dispersal on control and *Ecc15*-containing food in wild-type, *Gr33a*^*1*^*; UAS-Gr33a,* and *Gr33a*^*1*^*; Gr33a-GAL4 > UAS-Gr33a* genotypes. Statistical analyses were performed using a mixed-effects model (REML) with time and condition as fixed factors, (ns = not significant; *p < 0.05; ****p < 0.0001). Comparisons between selected conditions are shown. For all Figures, baseline behavioral characterization of parental GAL4 and UAS lines on *Ecc15*-contaminated food are provided in S17 Fig.

In *Drosophila*, non-volatile aversive cues are detected by gustatory receptor neurons (GRNs) expressing specific gustatory receptors (GRs) [35–37]. In larvae, the gustatory receptors Gr33a and Gr66a are co-expressed in bilateral pairs of GRNs located primarily in the terminal organ (TO) and in pharyngeal sensilla, which together constitute the core aversion-sensitive GRN population [38]. To evaluate their functional requirement in *Ecc15*-induced dispersal, Gr66a-positive neurons were silenced by overexpressing the inward-rectifier potassium channel Kir2.1 (*Gr66a-GAL4 > UAS-Kir2.1*), thereby hyperpolarizing these cells and preventing synaptic transmission [39]. Larvae with inactive Gr66a-positive neurons showed markedly reduced dispersal from *Ecc15-*contaminated food compared to controls, while their behavior on uncontaminated yeast remained unchanged (Fig 4A). A similar reduction in dispersal was observed following silencing of Gr33a-positive neurons (*Gr33a-GAL4 > UAS-Kir2.1*) (S9A Fig). Together, these findings indicate that aversion-sensitive GRNs are essential for the escape behavior triggered by *Ecc15* contamination.

In addition to Gr66a and Gr33a, Gr32a and Gr93a, two receptors involved in aversive contact behaviors, are expressed in partially overlapping subsets of aversion-sensitive GRNs [38,40–42]. To determine the specific contribution of each GR, loss-of-function mutant alleles for *Gr33a* (*Gr33a¹*), *Gr66a* (*Gr66a*^*ex83*^), *Gr32a* (*Gr32a*^*KO*^), and *Gr93a* (*Gr93a³*) were analyzed. *Gr66a*^*ex83*^ and *Gr32a*^*KO*^ larvae dispersed at wild-type levels from contaminated food, indicating that Gr66a and Gr32a are not required for *Ecc15*-induced dispersal (Fig 4B–4C). *Gr93a³* mutants showed heightened dispersal on contaminated food and mild nonspecific dispersal on uncontaminated yeast, suggesting that Gr93a modulates gustatory sensitivity rather than specifically mediating bacterial avoidance (Fig 4D). By contrast, *Gr33a¹* larvae no longer escape from *Ecc15*-contaminated yeast, and remain on the substrate throughout the assay (Fig 4E). This phenotype was not attributable to defects in attraction or locomotion: when positioned at a distance from a food source, *Gr33a¹* larvae readily oriented toward and reached both clean and contaminated substrates, but, unlike controls, failed to withdraw after contacting *Ecc15*-contaminated yeast (S9B Fig). Reintroducing *Gr33a* expression in Gr33 positive neurons (*Gr33a¹; Gr33a-GAL4 > UAS-Gr33a*) restored dispersal to wild-type levels (Fig 4F). Together, these results demonstrate that Gr33a plays a critical role in *Ecc15*-induced dispersal, highlighting its contribution to the contact-dependent detection of *Ecc15*-derived cues within the larval gustatory circuit.

### *orco–Or49a* olfactory and *Gr33a* gustatory circuits cooperate to mediate larval escape from *Ecc15*-contaminated food

**Fig 5:**
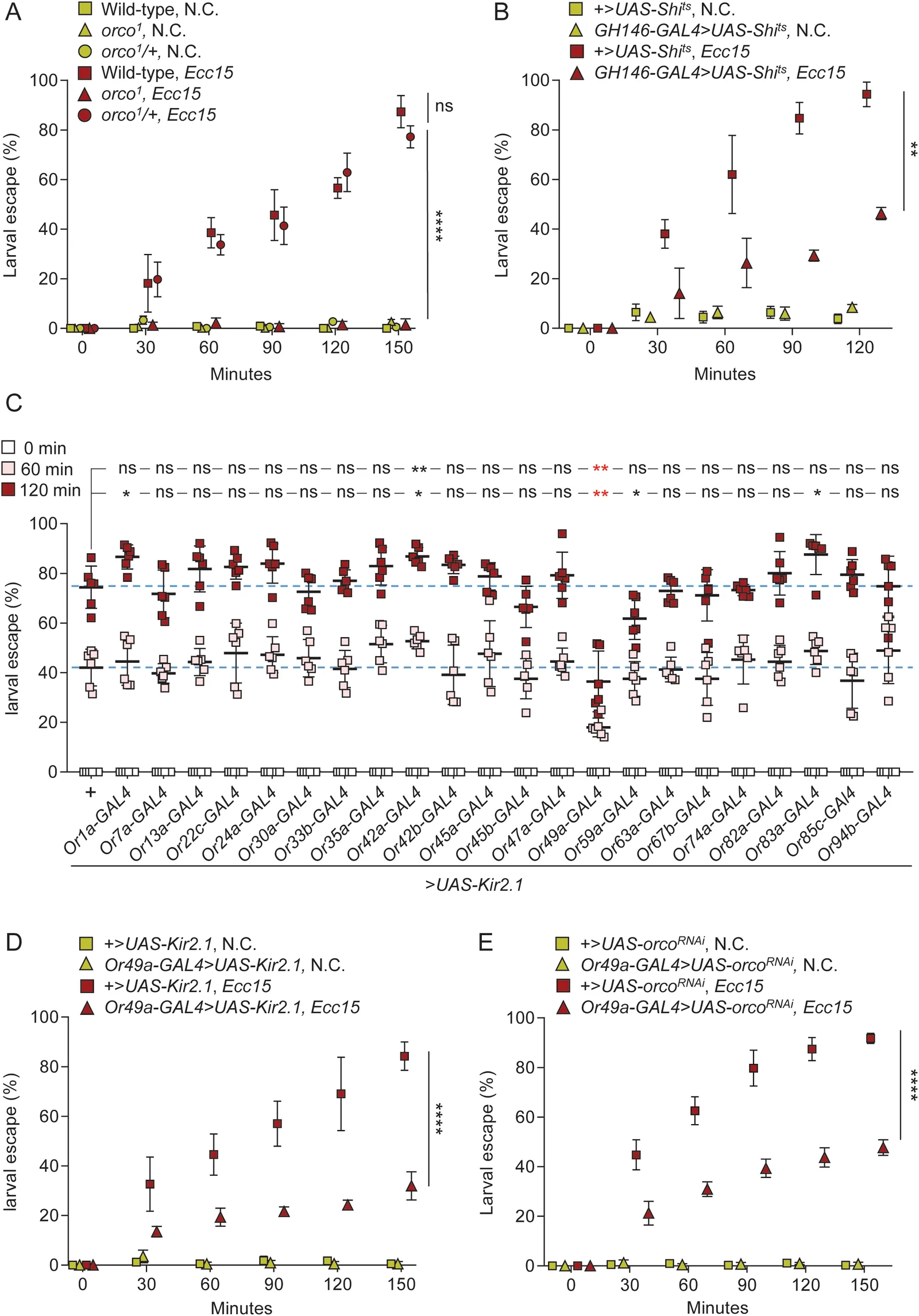
The orco–Or49a olfactory pathway mediates larval escape response to *Ecc15*-contaminated food. **(A)** Behavioral response of wild-type, *orco¹* mutant and *orco¹*/ + heterozygotes larvae on uncontaminated (N.C) and *Ecc15*-supplemented yeast. **(B)** Response of *GH146-GAL4 > UAS-Shi*^*ts*^ larvae to *Ecc15*-contaminated food at restrictive temperature (31°C). **(C)** Escape induced by *Ecc15* at 60 and 120 min after silencing individual larval olfactory neuron classes (*OrX-GAL4 > UAS-Kir2.1*)*.* Data are presented as means ± SD. Individual squares represent biological replicates (each consisting of groups of ≥ *40* larvae. **(D)** Larval dispersal of *Or49a-GAL4 > UAS-Kir2.1* larvae on uncontaminated or *Ecc15*-contaminated food. **(E)** Escape behavior of *Or49a-GAL4 > UAS-orco*^*RNAi*^ larvae on *Ecc15*-contaminated food. Statistical analyses were performed using a mixed-effects model (REML) with time and condition as fixed factors for panels A, B, D, and E (ns = not significant; **p < 0.01; ****p < 0.0001). Comparisons between selected conditions are shown. Panel C was analyzed using the Mann–Whitney test (ns = not significant; *p < 0.05; **p < 0.01).

Olfaction provides a critical sensory input that complements gustation in detecting aversive environmental cues [43]. To determine whether volatile compounds contribute to the dispersal behavior triggered by bacterial contamination, larvae carrying null mutations in the olfactory co-receptor *orco* (*orco¹* and *orco²*) were tested. While wild-type and heterozygous *orco¹/ +* larvae rapidly escaped from *Ecc15* contaminated substrate, anosmic *orco¹* mutants displayed a complete loss of this avoidance behavior (Fig 5A). Similar results were obtained with *orco²* mutants and *orco¹/orco²* transheterozygotes (S10A-S10B Fig). Consistently, blocking synaptic transmission in *orco*-expressing olfactory receptor neurons (ORNs) using the temperature-sensitive blocker *shibire*^*ts*^ [39] (*orco-GAL4 > UAS-shi*^*ts*^) markedly reduced dispersal from contaminated food (S10C–S10D Fig). These findings show that *orco*-dependent ORNs are required for detecting volatile cues that trigger larval escape.

To further delineate the olfactory circuitry, the role of projection neurons (PNs) transmitting sensory input from peripheral ORNs was examined. *orco*-positive ORNs project their axons to specific glomeruli within the larval antennal lobe, where they synapse onto GH146-GAL4-labeled PNs [44]. Silencing these neurons with *shibires*^*ts*^ strongly reduced dispersal on contaminated yeast, without affecting behavior on uncontaminated yeast, demonstrating that *orco*-dependent PNs are necessary for olfactory signal transmission driving dispersal (Fig 5B).

GH146-positive PNs send axons to two higher-order processing centers: the lateral horn (LH), which mediates innate olfactory responses, and the mushroom body (MB), involved in learning and multisensory integration [45,46]. Blocking synaptic transmission in Kenyon cells, the intrinsic MB neurons, using *OK107-GAL4 > UAS-shibire*^*ts*^ [47] had no effect on larval escape from contaminated food, suggesting that the MB is dispensable and that the rapid dispersal response relies on innate olfactory processing (S11 Fig).

Next, the role of individual odorant receptors (Ors) was investigated. The larval olfactory system contains 21 ORNs in the dorsal organ (DO), each expressing a specific Or together with Orco [48,49]. Selective inactivation of Orco-dependent ORN subsets via Kir2.1 overexpression (*OrX-GAL4 > UAS-Kir2.1*) showed that inhibition of Or49a-positive neurons specifically and reproducibly reduced dispersal, without affecting behavior on uncontaminated yeast (Fig 5C–5D). To rule out locomotor or orientation deficits, *Or49a-GAL4 > UAS-Kir2.1* larvae were placed away from the food source; they oriented toward both uncontaminated and contaminated yeast but exhibited reduced escape frequency after contact with contaminated food, consistent with impaired escape initiation (S12 Fig).

*Or49a* expression was previously reported in one DO neuron co-expressing Orco and one TO neuron [48,49] (S13A Fig). Targeted knockdown of *orco* in Or49a-expressing neurons (*Or49a-GAL4 > UAS-orco*^*RNAi*^) significantly decreased dispersal, indicating that Or49a-dependent olfactory signaling contributes to the detection of volatile bacterial cues required for escape (Fig 5E). Given that Or49a is also expressed in the TO, overlap with known aversive gustatory neuron populations was examined. Double-driver labeling (*Or49a-GAL4; Gr33a-GAL4 > UAS-GFP*) revealed one additional TO neuron compared to *Gr33a-GAL4 > UAS-GFP* controls alone, demonstrating that *Or49a-GAL4* and *Gr33a-GAL4* label distinct neuronal populations (S13A Fig). This observation was further supported by dual-reporter analysis using *Or49a-GAL4 > UAS-GFP* and *Gr66a-LexA > LexAOP-mCherry*, which showed no co-localization between reporter signals (S13B Fig). Together, these findings indicate that Or49a-GAL4-positive neurons are distinct from known aversive gustatory populations, while the contribution of the TO Or49a neuron to escape behavior remains unresolved.

To gain further insight into the functional relationship between the olfactory (Or49a–Orco) and gustatory (Gr33a) pathways, genetic interaction analysis was performed in *Gr33a¹/ + ; orco¹/ +* larvae. These double heterozygotes displayed escape behavior comparable to that of wild‑type and single heterozygous controls (S14 Fig). Accordingly, the results do not support a simple combinatorial threshold model in which the two pathways contribute additively.

Altogether, these findings reveal that larval escape from *Ecc15*-contaminated food depends on a cooperative multisensory network combining gustatory and olfactory inputs: *Gr33a*-dependent neurons detect contact-dependent bacterial cues, while Or49a–Orco signaling mediates perception of volatile compounds, jointly driving rapid dispersal.

### Larval behavioral response to *Ecc15*-contaminated food modulates survival, development, and pathogen dissemination

The preceding results show that larval escape from *Ecc15*-contaminated substrates is triggered by the combined action of gustatory and olfactory cues upon contact with the food source. This behavior likely reduces the duration of exposure to contaminated substrates, raising the question of its consequences for larval survival and development. To address this, second-instar larvae were placed on uncontaminated yeast or yeast contaminated with *Ecc15* or *E. coli*, and their growth and developmental progression were monitored.

**Fig 6:**
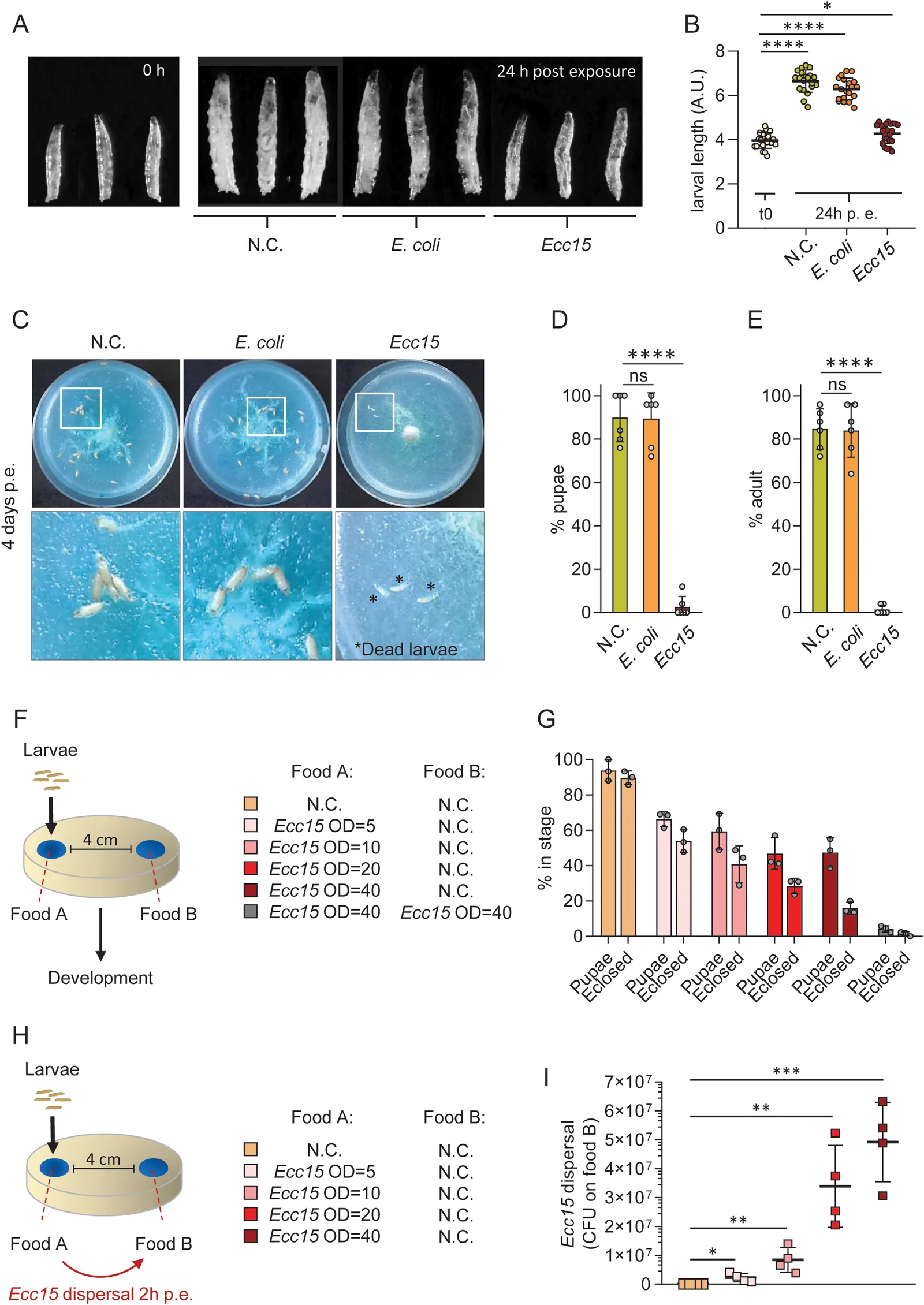
Exposure to *Ecc15*-contaminated food impairs larval development and promotes bacterial dissemination. **(A)** Representative images of larvae at 0 h and 24 h after exposure to control food (N.C.) or food supplemented with *E. coli* or *Ecc15*. **(B)** Quantification of larval body lengths at 0 h and 24 h following exposure to control or contaminated food in arbitrary units (A.U.). Data are presented as means ± SD. Individual points represent individual larvae (n ≥ 20)*.* **(C)** Representative images illustrating pupal formation four days after larval exposure to control or contaminated food; boxed regions are shown at higher magnification below. **(D–E)** Proportion of larvae that reached the pupal stage after four days (D) or eclosed as adults after seven days (E) following exposure to control or contaminated food. Data are presented as means ± SD. Individual points represent biological replicates (each consisting of *25* larvae). **(F)** Schematic of the assay used to evaluate developmental outcomes of larvae that relocate to uncontaminated food after an initial exposure to *Ecc15*-contaminated substrate. **(G)** Proportion of larvae exposed to increasing concentrations of *Ecc15* (OD_600_ = 5, 10, 20, and 40) that reached the pupal stage or eclosed following relocation to an uncontaminated food source (N.C.). Data are presented as means ± SD. Individual points represent biological replicates (each consisting of groups of *≥* 35 larvae). **(H)** Schematic of the bacterial dissemination assay. **(I)** *Ecc15* retrieved (CFU) from initially uncontaminated food (N.C) two hours after larvae were exposed to contaminated substrate. Data are presented as means ± SD. Individual squares represent biological replicates. Statistical analyses were performed using the Mann–Whitney test for panel B and Fisher’s exact test for panels D and E (ns = not significant, *p < 0.05; ****p < 0.0001). Panel G was analyzed using Chi-square tests that demonstrate that *Ecc15* concentration impacts on the pupation of larvae and on emergence of adult flies (****p < 0.0001). Panel I was analyzed using unpaired t test (*p < 0.05; **p < 0.01; ***p < 0.001).

After 24 h, larvae maintained on uncontaminated or *E. coli*–contaminated food exhibited comparable growth (Fig 6A–6B) and progressed normally through development, with similar pupation and adult emergence rates, consistent with their ability to thrive on substrates enriched with non-pathogenic bacteria (Figs 6C–6E and S15). In contrast, larvae exposed to *Ecc15* left the contaminated substrate, showed no significant growth (Fig 6A–6B), and rarely pupated, with almost no adult emergence (Figs 6C–6E and S15). These observations indicate that exposure to *Ecc15* triggers dispersal and persistent avoidance, while restrictive conditions that prevent relocation lead to developmental arrest and high mortality.

To determine whether access to alternative resources mitigates the developmental consequences of *Ecc15* exposure, larvae were placed on uncontaminated yeast or yeast contaminated at increasing *Ecc15* bacterial loads, with each yeast patch positioned at a distance from a second, uncontaminated food source (Fig 6F). After 24 hours, larvae initially placed on uncontaminated yeast largely remained on the original patch, indicating limited dispersal in the absence of pathogen cues (S16A–S16B Fig). In comparison, larvae exposed to *Ecc15* consistently relocated to the uncontaminated patch at all bacterial concentrations (S16B Fig). Despite this relocation, developmental analysis revealed a dose-dependent impairment, with pupation and eclosion rates significantly lower than those of larvae initially placed on uncontaminated food (Fig 6G). Thus, access to an uncontaminated refuge partially alleviates, but does not fully prevent, the detrimental effects of initial *Ecc15* exposure.

Given the robust dispersal behavior triggered by *Ecc15*, including relocation to uncontaminated substrates, the possibility that larvae contribute to bacterial dissemination was next examined. Larvae were placed on contaminated yeast at increasing bacterial loads in the same two-patch assay configuration (Fig 6H). Within two hours, larvae were consistently detected on the clean substrate, with the number of relocated individuals increasing with the initial bacterial load (S16C Fig). Viable *Ecc15* cells were recovered from these previously uncontaminated patches, with abundance correlating with both the number of migrating larvae and the initial bacterial load (Fig 6I).

Together, these findings show that the escape response elicited by *Ecc15*-contaminated food, characterized by initial contact followed by rapid withdrawal and relocation, reduces direct exposure to contaminated substrates yet fails to fully prevent detrimental developmental consequences. At the same time, this behavior promotes the transfer of viable bacteria to new food sources, indicating that larval *Drosophila* can contribute to the environmental dissemination of *Ecc15*.

## Discussion

Animals developing in microbe-rich environments are constantly exposed to microbial cues and can display behavioral responses upon encountering contaminated substrates [19,50,51]. Here, we show that *Drosophila melanogaster* larvae exhibit a rapid, robust escape behavior when encountering food contaminated with *Ecc15*. This response, which has not been previously described for this natural pathogen, expands the repertoire of known larval behavioral avoidance and highlights the importance of ecological context in shaping larval responses to microbial cues.

### Larval escape is triggered under specific ecological and bacterial metabolic conditions

Behavioral responses to pathogens have been far less explored in *Drosophila* larvae than in adults. Previous work showed that larvae can abandon food contaminated with *P. entomophila*, but this response emerges over several hours and is strongly influenced by the physiological state of the animal. In particular, starvation reduces avoidance behavior, indicating that internal nutritional cues modulate pathogen-induced responses. In that study, no avoidance response to *Ecc15* was detected under the conditions tested [26].

The escape behavior described here differs in several key respects. It is rapidly initiated, within minutes after contact with *Ecc15*-contaminated food, and comparable dispersal occurs in both fed and starved larvae. Together, these differences indicate that the response observed in this study represents a distinct form of behavioral avoidance.

In the present work, larval escape was found to depend on the physiological state of *Ecc15*. It occurred only when stationary-phase cells were provided with access to nutrients permitting metabolic reactivation, while bacterial numbers remain stable, ruling out proliferation as the trigger. Thus, escape appears to rely on bacterial metabolic activity rather than abundance, highlighting the importance of bacterial physiological state in host detectability and behavioral impact. The activity was retained for several days on the substrate, suggesting that bacterial metabolic activation leads to the production of behaviorally active, as yet unidentified cues that remain stable in the food environment and can drive behavior independently of ongoing growth.

Importantly, under the conditions tested here, escape is observed with *Ecc15* but not with other bacterial species tested, including *E. coli*, *P. entomophila*, and *L. brevis*. This suggests that larval escape is not triggered by bacterial presence in a generic manner, but rather by specific bacterial signals associated with *Ecc15* under conditions permissive for metabolic activity. Whether other bacteria relevant to *Drosophila* ecology, including additional *Erwinia* species [6], can also trigger rapid escape remains an open question. More broadly, the widespread coupling between bacterial metabolic activity and production of extracellular factors during growth suggests that similar mechanisms may underlie cue generation beyond *Ecc15*.

### Larval escape relies on complementary gustatory and olfactory cues

Our findings reveal that larval escape from *Ecc15* relies on the cooperation of two distinct chemosensory modalities: gustation and olfaction. To our knowledge, this is the first demonstration of a rapid, multisensory escape behavior triggered by a natural pathogen in *Drosophila* larvae. The gustatory receptor Gr33a, a broad aversion receptor associated with bitter and toxic compound detection [36,40–42], is essential for the response. Although Gr33a often functions together with Gr66a in adult taste circuits, our data indicate that Gr33a acts here independently of Gr66a, pointing to a Gr33a-dependent mechanism of danger detection. The requirement for contact-dependent gustatory input suggests that bacterial metabolic activity produces at least one non-volatile cue capable of triggering Gr33a-dependent larval escape.

In parallel, an olfactory component is required. The odorant receptor Or49a, functioning together with the co-receptor Orco and previously implicated in the detection of the parasitoid wasp pheromone iridomyrmecin [52], is also critical for dispersal. This finding indicates that the Or49a‑Orco olfactory receptor complex is required for detecting a volatile cue produced by *Ecc15*, distinct from iridomyrmecin, and highlights its functional versatility. The involvement of Or49a aligns with growing evidence that the larval olfactory system, though anatomically simple, contains dedicated circuits for detecting ecologically salient threats [52,53].

Strikingly, disruption of either the gustatory or the olfactory pathway abolishes escape, indicating that both sensory modalities are required for behavioral output. Volatile and contact-dependent cues therefore likely provide complementary information about bacterial contamination. Such multisensory processing may improve substrate evaluation by relying on both volatile and contact-dependent information associated with contaminated substrates. Whether these cues correspond to different chemical forms of the same compound or to distinct metabolites remains unknown.

Two models can account for this dual requirement. In a convergence model, gustatory and olfactory pathways interact within shared downstream circuits to jointly drive the response. In a threshold model, each pathway contributes independently to a cumulative signal, such that loss of either input reduces that signal below the threshold needed to trigger escape. Consistent with the involvement of distinct sensory populations, no overlap was detected between Gr33a- and Or49a-expressing neurons. Moreover, in *Gr33a¹/ + ; orco¹/ +* double heterozygous larvae, no enhanced behavioral deficit was found relative to single heterozygotes or wild-type controls. Together, these results are more consistent with circuit-level interdependence or partial functional coupling than with a strict additive threshold mechanism. However, the genetic perturbations used here likely reduce rather than abolish sensory activity, precluding a definitive mechanistic distinction between circuit-level convergence and broader functional cooperation.

Although the circuit-level relationship between gustatory and olfactory inputs remains unresolved, neuronal manipulations provide insight into the broader circuit organization underlying escape behavior. Silencing GH146-positive projection neurons, which innervate the larval antennal lobe and relay olfactory information to higher brain centers, indicated that escape depends on olfactory projection pathways [45,53]. These neurons project to both the LH and MB [54–56], yet silencing Kenyon cells had no effect, suggesting that associative MB circuits are not required. Taste information from Gr33a-expressing neurons is relayed to the subesophageal zone (SEZ), the primary gustatory processing center in larvae [38,40,42]. While SEZ output pathways remain incompletely mapped, they clearly connect gustatory processing to higher-order protocerebral regions.

The combined requirement for GH146-positive projection neurons and Gr33a-expressing neurons, together with the rapid, stereotyped, and decision-like nature of the behavior, supports the view that escape is likely routed through non-associative circuits consistent with innate valence pathways. However, the precise circuit locus underlying the joint contribution of these pathways remains to be determined. At a broader anatomical level, this interpretation aligns with the established role of the LH in mediating hardwired responses to ecologically salient stimuli, whereas the MB is generally required for learned aversions [46,57].

These findings further establish this behavioral paradigm as a tractable model for dissecting how gustatory and olfactory cues cooperate to guide ecologically relevant avoidance responses. The genetic accessibility of the larva, together with the ability to manipulate both sensory modalities independently, makes this system well suited for mapping the cellular and circuit mechanisms underlying the translation of bacterial-derived cues into behavior.

### Larval escape reduces local exposure while promoting pathogen dissemination

Larval escape from *Ecc15*-contaminated food promotes relocation toward uncontaminated resources, thereby reducing the duration of contact with contaminated substrates. Developmental analyses nevertheless indicate that this response provides only partial mitigation of the detrimental effects associated with *Ecc15* exposure. Although access to an uncontaminated refuge improved pupation and eclosion relative to larvae maintained under restrictive conditions without alternative food sources, developmental success remains substantially impaired compared to uncontaminated controls. These findings indicate that escape alters exposure dynamics without fully preventing the deleterious consequences associated with *Ecc15* exposure. More generally, they suggest that the outcome of avoidance behavior depends critically on environmental spatial organization and resource heterogeneity.

Importantly, these experiments were performed under simplified laboratory conditions that likely differ from the ecological complexity of fermenting substrates. In natural environments, bacterial density, microbial community composition, substrate turnover, and food distribution would all be expected to influence both exposure dynamics and the effectiveness of larval relocation. Under more heterogeneous ecological conditions, rapid withdrawal from contaminated substrates could therefore contribute more substantially to reducing exposure to harmful microbes.

Escape behavior also promotes bacterial dissemination. Dispersing larvae transferred viable *Ecc15* cells to previously uncontaminated food patches, indicating that larval movement contributes directly to bacterial spread under the conditions examined here. Thus, the same behavior that reduces local exposure to contaminated food also enhances the spatial redistribution of viable bacteria across food resources. Comparable forms of insect-mediated bacterial dissemination are well documented in the ecology of plant-associated microbes, where host mobility contributes substantially to bacterial transmission between substrates [58]. In this context, the ability of *Drosophila* adults to disseminate *Erwinia* species in natural environments raises the possibility that both larval and adult stages participate in the environmental circulation of these bacteria [11,12].

Together, these observations suggest that larval escape generates dual ecological consequences for both host and bacterium. While relocation reduces contact with contaminated food, larval movement concurrently redistributes viable *Ecc15* cells across food patches. Rather than reflecting an outcome that is exclusively beneficial to either partner, this behavioral response may therefore represent an ecological interaction in which host avoidance indirectly contributes to bacterial dissemination. Escape, triggered under conditions that support bacterial metabolic activity, likely reflects the sufficiency of growth-associated bacterial cues to induce larval relocation.

### Conclusions and perspectives

This study identifies a rapid escape behavior in *Drosophila* larvae triggered by the natural pathogen *Ecc15*. This response is selective, requiring metabolically active bacteria and engaging complementary gustatory (Gr33a-dependent) and olfactory (Orco–Or49a-dependent) pathways. Together, these sensory inputs enable larvae to detect contaminated food sources and rapidly relocate to alternative resources when available.

Beyond its sensory basis, this behavior also has important ecological consequences. While dispersal can reduce prolonged exposure to contaminated substrates under spatially heterogeneous conditions, it also promotes the transfer of viable bacteria to new food patches. These findings therefore highlight how a single host avoidance behavior can reshape both host exposure and microbial spatial distribution.

More broadly, this work establishes larval escape from *Ecc15* as a tractable model for understanding how microbial physiological states shape multisensory behavioral responses and ecological interactions. This system provides a framework for investigating how neural processing and environmental context jointly influence host–microbe interactions.

## Materials and methods

### Fly stocks and husbandry

The following *Drosophila melanogaster* strains were used in this study. The *w*^*1118*^ strain (no. 5905, Bloomington *Drosophila* Stock Center, BDSC) served as the reference wild-type strain in all main experiments unless otherwise indicated. Additional lines obtained from the BDSC included: *Canton-S* (no. 64349), *Oregon-R* (no. 25211), *PGRP-LE*^*112*^ (no. 33055), *orco*^*1*^ (no. 23129), *orco*^*2*^ (no. 23130), *GH146-GAL4* (no. 91812), *OK107-GAL4* (no. 854), *UAS-Shibire*^*ts*^ (no. 44222), *UAS-Kir2.1* (no. 6595), *Gr66a-GAL4* (no. 28801), *Gr33a-GAL4* (no. 57624), *Orco-GAL4* (no. 23292), *UAS-mCD4-Tomato* (no. 35841), *Gr66a*^*ex83*^ (no. 25027), *Gr33a*^*1*^ (no. 31427), *Gr93a*^*3*^ (no. 27592), *Gr33a*^*1*^; *Gr33a-GAL4* (no. 31425), *Gr33a*^*1*^*; UAS-Gr33a-GAL4* (no. 31424), *Or1a-GAL4* (no. 9949), *Or7a-GAL4* (no. 23907), *Or13a-GAL4* (no. 9945), *Or22c-GAL4* (no. 9953), *Or24a-GAL4* (no. 9957), *Or30a-GAL4* (no. 9960), *Or33b-GAL4* (no. 9964), *Or35a-GAL4* (no. 9968), *Or42a-GAL4* (no. 9972), *Or42b-GAL4* (no. 9976), *Or47a-GAL4* (no. 9982), *Or49a-GAL4* (no. 9985), *Or59a-GAL4* (no. 9989), *Or63a-GAL4* (no. 9991), *Or67b-GAL4* (no. 9996), *Or74a-GAL4* (no. 23124), *Or82a-GAL4* (no. 23125), *Or83a-GAL4* (no. 23127), *Or85c-GAL4* (no. 23914), *Or94b-GAL4* (no. 23145), *TRPA1* (no. 26504) and *UAS-6xGFP* (no. 52262). The *yellow white* reference strain was kindly provided by Dr. B. Charroux (Aix-Marseille University, France). The *Dredd*^*D55*^ and *Gr32a*^*KO*^ mutant strains were generous gifts from Dr. B. Lemaire (EPFL, Switzerland) and Dr. J.R. Carlson (Yale University, USA), respectively. The *UAS-Orco*^*RNAi*^ line (KK100825) was obtained from the Vienna *Drosophila* Resource Center (VDRC). The *PGRP-LC*^*E12*^ mutant was described previously [59]. The *Gr66a-LexA*; *LexAop-mCherry* line was kindly provided by Dr. Yali V. Zhang (University of Pennsylvania, USA).

Flies were maintained at 25 °C on standard yeast/cornmeal medium under a 12 h light/12 h dark cycle. For 1 L of food, 8.2 g agar (VWR), 80 g cornmeal (Westhove Farigel maize H1), and 80 g yeast extract (VWR) were boiled for 10 min, cooled, and supplemented with 5.2 g methylparaben sodium salt (MERCK) and 4 mL 99% propionic acid (CARLO ERBA).

### Preparation and inactivation of bacterial and yeast cultures

The bacterial strains used included rifampicin-resistant *Erwinia carotovora carotovora 15* (*Ecc15*) [6], *Ecc15* Δ*evf* [29], *Ecc15* Δ*4* [30], as well as *Escherichia coli* K-12 M4100, *Pseudomonas entomophila*, and *Levilactobacillus brevis*, kindly provided by Dr. I. Gomperts Boneca (Institut Pasteur, France), Dr. A. Gallet (CNRS, INRAE, France), and Dr. F. Leulier (CNRS, France), respectively.

Single colonies were used to inoculate 250 mL of culture medium. *Ecc15* and *P. entomophila* were grown in LB broth (Lennox formulation, Invitrogen, cat. L22897) at 30 °C under agitation (250 rpm), whereas *E. coli* was cultured in LB at 37 °C under the same conditions. *L. brevis* was grown in MRS broth (Sigma-Aldrich, cat. 69966) at 37 °C under static, anaerobic-like conditions. Bacterial growth was monitored by OD_600_. Cultures were centrifuged at 3800 rpm for 15 min, and pellets were resuspended in PBS to OD_600_ = 200 before mixing with baker’s yeast to obtain the desired final OD_600_.

To establish the correspondence between OD₆₀₀ values and bacterial load in yeast–bacteria mixtures, yeast–*Ecc15* samples were prepared at defined final OD₆₀₀ values (5, 10, 20, and 40). Aliquots (100 µL) of these mixtures were homogenized in 1 mL PBS, and colony-forming units (CFUs) were determined by serial dilution and plating on LB agar supplemented with rifampicin, followed by overnight incubation at 30 °C.

Heat-killed *Ecc15* was obtained by incubating pellets at 95 °C for 10 min followed by rapid cooling at −20 °C. Baker’s yeast (*S. cerevisiae*) was inactivated at 80 °C for 10–15 min and cooled to room temperature. Heat-killed bacteria and inactivated yeast were processed identically to live cells in subsequent assays.

### Behavioral assays

A 100 µL bacterial suspension was mixed with 0.5 g commercial baker’s yeast (~0.5 mL) or, when indicated, with 0.5 g mashed ripe banana (~0.5 mL). Final OD_600_ was calculated as: Final OD_600_ = 100 μL × Initial OD_600_/ 500 μL. A 100 µL drop of food–bacteria mixture was placed on LB agar plates and incubated for 30 min, unless otherwise specified. Second-instar larvae (48 h at 25°C after egg laying) were washed in water and tested in groups of n ≥ 40 per replicate, unless otherwise stated. Each experiment included at least three technical replicates and was independently repeated three times or more.

In dispersal assays, larvae were placed directly on food; in avoidance and binary choice assays, larvae were positioned 4 cm from the food source(s). At each time point, larvae outside the food were counted under a ZEISS Stemi 508 stereomicroscope. At the end of each assay, the food was suspended in water and larvae counted after liquid removal. Escape percentages were calculated relative to the initial number of larvae.

Behavioral assays were performed at room temperature, unless otherwise specified, in darkness; illumination was applied only briefly during counting to minimize phototactic bias.

### Imaging of larval behavior

For time-point imaging, groups of >100 larvae were placed directly on the food source. Experiments were conducted in darkness, and white light was applied only briefly for image capture. Images were acquired using a Logitech HD Pro Webcam C920.

For movie generation, time-lapse imaging was performed using the same setup, controlled by Yawcam v0.8.0, capturing one frame every 2 s over 3 h 30 min. During acquisition, white-light intensity and direction were minimized to reduce phototactic responses. Image sequences were compiled into movies using Fiji (ImageJ v1.54p) and rendered at 24 fps. All images, including those used for time-point photography and movie generation, were processed with Photoshop CS6.

### Larval growth, development, and survival

Second-instar larvae were placed on control yeast or yeast mixed with *Ecc15* or *E. coli*. After 24 h, larvae were washed in phosphate-buffered saline (PBS), fixed in 70% ethanol, mounted, and imaged using a Zeiss Discovery Lumar V12 microscope. The size of ≥20 larvae per condition was quantified in arbitrary units using Fiji (ImageJ v1.54g) and compared to unexposed second instar larvae.

For development and survival assays, groups of 25 second instar larvae per condition were monitored for pupation (day 4) and adult eclosion (day 7) following exposure to the food source. Counting was performed using a ZEISS Stemi 508 stereomicroscope, and images were acquired with a Logitech HD Pro Webcam C920.

### Assessment of bacterial dissemination by *Drosophila* larvae

To evaluate bacterial transfer from contaminated to uncontaminated food sources, groups of 25 larvae were placed on yeast mixed with *Ecc15* (OD₆₀₀ = 5–40), located 4 cm from uncontaminated yeast. All conditions were performed in four technical replicates. After 2 h, the proportion of larvae that reached the uncontaminated yeast was quantified. The uncontaminated food was collected and homogenized in 1 mL PBS. Colony-forming units (CFUs) were determined by serial dilution of the homogenate and plating on LB agar supplemented with rifampicin, followed by overnight incubation at 30 °C.

### Bacterial components and culture media

Ultrapure peptidoglycan from *E. coli* (PGN-ECndi; InvivoGen, USA, cat. Tlrl-kipgn) and lipopolysaccharides (LPS) from *E. coli* O111: B4 (Sigma-Aldrich, cat. L2630) were used as bacterial components in experimental assays.

For the preparation of solid media, Luria–Bertani (LB) broth (Lennox formulation, Invitrogen, cat. L22897), agar (BD Bacto, Becton Dickinson, cat. 214010), high-purity agarose (NEEO Ultra, Euromedex, cat. 2267.4), and commercial apple juice were used. Apple agar consisted of apple juice supplemented with agar and 0.005% Tween-20 to improve surface wetting and promote uniform contact between the yeast–bacteria mixture and the substrate. All reagents were handled according to the manufacturers’ instructions.

### Confocal imaging

Second instar larvae of the specified genotypes were heat-killed (60 °C, 10 s), mounted in Vectashield (Vector Labs, Cat. No. H-1200), and imaged using a Zeiss LSM 780 confocal microscope with a 20 × air objective. Images were processed using Adobe Photoshop CS6.

### Data representation and statistical analyses

All graphical representations and statistical analyses were performed using GraphPad Prism 8. Larval escape dynamics were analyzed using a mixed-effects model (REML) with Time and Condition as fixed factors, including their interaction. The T0 time point was excluded because all conditions are identical at this stage. Post-hoc multiple comparisons were carried out using Tukey’s test. For all experiments, full raw datasets and complete statistical outputs are provided in S1 and S2 Data.

## Supporting information

S1 Fig Growth dynamics and CFU-based quantification of *Ecc15* under different conditions. (A) Growth curve of *Ecc15* cultured in nutrient-rich LB under shaking conditions at 30 °C. (B) Growth or survival dynamics of stationary-phase *Ecc15* resuspended in fresh LB or PBS and maintained in static culture at room temperature (RT). (C) Corresponding CFU counts of stationary-phase *Ecc15* in yeast–bacteria mixtures prepared at final OD₆₀₀ values of *Ecc15* (5, 10, 20, and 40). CFU counts were determined by serial dilution and plating on LB agar supplemented with rifampicin. Data are means ± SD (n = 5). (D) CFU counts of *Ecc15* recovered from the same yeast–bacteria mixtures as in (C), measured immediately after deposition on LB agar (0 min) or after 30 min at RT. Pairwise comparisons between 0 min and 30 min for each OD were performed using an unpaired t-test (ns, not significant).
(PDF)

S2 Fig *Ecc15* triggers a sustained and dose-dependent escape behavior in *Drosophila* larvae. Quantification of second-instar larvae (*w¹¹¹⁸*) escape behavior from uncontaminated yeast (N.C.) or yeast contaminated with increasing concentrations of *Ecc15* (OD_600_ = 5, 10, 20, and 40) at multiple time points. For this and all subsequent supplementary figures, dispersal assay results are presented as mean ± SD of technical triplicates from a representative biological experiment. Each experiment was independently repeated at least three times with groups of ≥40 larvae per replicate, unless otherwise indicated. Statistical analyses were performed using two-way repeated measures ANOVA, followed by Tukey’s multiple comparisons test (ns = not significant; *p < 0.05; **p < 0.01; ***p < 0.001; ****p < 0.0001). Comparisons between selected conditions are shown.
(PDF)

S3 Fig *Ecc15*-induced larval escape behavior is conserved across *Drosophila* melanogaster genetic backgrounds. Quantification of dispersal of second-instar larvae from *Canton-S*, *Oregon-R*, and *yellow white* strains on either uncontaminated yeast (N.C.) or yeast containing *Ecc15* or *E. coli* at identical bacterial densities (OD_600_ = 40). Statistical analyses were performed using a mixed-effects model (REML) with time and condition as fixed factors (ns = not significant; ****p < 0.0001). Pairwise comparisons between selected conditions are shown.
(PDF)

S4 Fig *Ecc15*-induced larval dispersal behavior is long-lasting and robust. Time-course quantification of second-instar *Drosophila* larvae (*w¹¹¹⁸*) escape behavior on freshly prepared *Ecc15*-contaminated yeast (OD₆₀₀ = 40) and on yeast–*Ecc15* mixtures maintained on LB agar plates at room temperature for 2, 4, or 6 days. Statistical analyses were performed using a mixed-effects model (REML) with time and condition as fixed factors (****p < 0.0001). Pairwise comparisons between uncontaminated and *Ecc15*-contaminated conditions are shown.
(PDF)

S5 Fig Time-course images of wild-type larvae on uncontaminated or bacterially contaminated yeast. Representative images of second-instar larvae (*w¹¹¹⁸*) at multiple time points, showing their positioning on either uncontaminated yeast (N.C.) or yeast contaminated with *Ecc15*, *E. coli*, *P. entomophila*, or *L. brevis* (OD₆₀₀ = 40). Yeast patches were deposited on nutrient-rich LB agar plates and incubated for 30 minutes at room temperature before imaging. In each condition, > 100 larvae were placed on the food source at t = 0. Images were captured with a standard webcam.
(PDF)

S6 Fig Larval dispersal from live *Ecc15* is independent of yeast metabolic state. Larval behavior on yeast–*Ecc15* mixtures prepared with metabolically active or heat-inactivated yeast. Statistical analyses used a mixed-effects model (REML) with time and yeast condition as fixed factors (*p < 0.05).
(PDF)

S7 Fig *Drosophila* larvae escape from *Ecc15*-contaminated ripe banana. Comparison of larval dispersal on uncontaminated ripe banana versus ripe banana contaminated with *Ecc15* (OD₆₀₀ = 40). Statistical analyses were performed using a mixed-effects model (REML) including time and condition as fixed factors. Statistical comparisons between non-contaminated and contaminated conditions are shown (****p < 0.0001).
(PDF)

S8 Fig Nutrient availability in the substrate modulates *Ecc15*-induced larval dispersal. Representative time-course images of second-instar larvae (*w¹¹¹⁸*) on yeast–*Ecc15* mixtures (OD₆₀₀ = 40) placed on substrates with different nutrient content: nutrient-rich LB agar, carbohydrate-rich but nitrogen-limited apple agar, low-nutrient agar, and nutrient-free agarose. In each condition, > 100 larvae were introduced onto the food at t = 0. Images were captured using a standard webcam.
(PDF)

S9 Fig Silencing the *Gr33a* neuronal circuit suppresses *Ecc15*-induced escape without altering locomotion or attraction to nutritive cues. (A) Dispersal in control larvae *(+>UAS-Kir2.1*) and in larvae with silenced Gr33a-expressing neurons (*Gr33a-GAL4 > UAS-Kir2.1*) on uncontaminated yeast (N.C.) or *Ecc15*-contaminated yeast. (B) Time-course quantification of wild-type and *Gr33a¹* mutant larvae located outside the food source when placed at a fixed distance from uncontaminated or *Ecc15*-contaminated yeast. Statistical analyses were performed using a mixed-effects model (REML) with time and condition as fixed factors; followed by Tukey’s post-hoc for-panel B comparing wild-type and *Gr33a¹* mutant larvae on *Ecc15*-contaminated food (ns = not significant; ****p < 0.0001).
(PDF)

S10 Fig The odorant co-receptor Orco is required for larval escape from *Ecc15*-contaminated food. (A–B) Time-course quantification of the escape behavior of wild-type, *orco²* mutant (A), and *orco¹/orco²* transheterozygous larvae (B) on uncontaminated (N.C.) or *Ecc15*-contaminated yeast. (C) Confocal images showing, from left to right: bright-field view of dorsal organs (DOs, arrows); *Orco-GAL4 > UAS-mCD4-Tomato* expression in olfactory receptor neurons (ORNs, arrows); merged image. (D) Escape behavior of control *(+>UAS-Shi*^*ts*^) and *Orco-GAL4 > UAS-Shi*^*ts*^ larvae exposed to either uncontaminated or *Ecc15*-contaminated yeast at the restrictive temperature (31 °C). Statistical analyses were performed using a mixed-effects model (REML) with time and condition as fixed factors (****p < 0.0001). Pairwise comparisons between contaminated conditions are shown.
(PDF)

S11 Fig Inactivation of mushroom body intrinsic neurons does not affect larval escape from *Ecc15*-contaminated food. Escape behavior of control *(+>UAS-Shi*^*ts*^) and *OK107-GAL4 > UAS-Shi*^*ts*^ larvae exposed to uncontaminated (N.C.) or *Ecc15*-contaminated yeast at the restrictive temperature (31 °C). Statistical analyses were performed using a mixed-effects model (REML) with time and condition as fixed factors (ns = not significant). Pairwise comparisons between contaminated conditions are shown.
(PDF)

S12 Fig Silencing Or49a-positive neurons reduces *Ecc15*-induced dispersal without affecting locomotion or attraction to nutritive cues. Quantification of the proportion of larvae located outside the food source over time in control larvae (*+>UAS-Kir2.1*) and those in which *Or49a-*expressing neurons were silenced (*Or49a-GAL4 > UAS-Kir2.1*). Larvae were placed at a fixed distance from either uncontaminated yeast or yeast contaminated with *Ecc15*. Statistical analyses were performed using a mixed-effects model (REML) with time and condition as fixed factors, followed by Tukey’s post-hoc tests (ns = not significant; *p < 0.05; ****p < 0.0001). Pairwise comparisons between *+>UAS-Kir2.1* and *Or49a-GAL4 > UAS-Kir2.1* larvae exposed to food contaminated with *Ecc15* are presented.
(PDF)

S13 Fig Non-overlapping expression of Or49a with Gr33a and Gr66a in larval sensory organs. (A) Confocal images showing GFP expression driven by the indicated GAL4 lines (left) and merged fluorescent and bright-field images (right) of the dorsal and terminal organs (DO and TO; arrows). Top: *Or49a-GAL4 > UAS-6xGFP*. Middle: *Gr33a-GAL4 > UAS-6xGFP*. Bottom: *Or49a-GAL4; Gr33a-GAL4 > UAS-6xGFP*. Asterisks indicate neuronal cell bodies. Double-driver labeling revealed one additional TO neuron compared to Gr33a-GAL4 > UAS-6xGFP alone. (B) Confocal images showing, from left to right, GFP expression driven by *Or49a-GAL4 > UAS-6xGFP*, mCherry expression driven by *Gr66a-LexA > LexAOP-mCherry*, merged fluorescent images, and corresponding merged images overlaid with bright-field (TO; arrow). No overlap between Or49a- and Gr66a-expressing neurons was observed. Scale bars: 20 μm.
(PDF)

S14 Fig Double heterozygous *Gr33a*^*1*^*/* *+* *; orco*^*1*^*/* *+* larvae retain normal escape response to *Ecc15.* Behavioral response of wild-type, *Gr33a¹/ +* , *orco¹/ +* heterozygous larvae, and *Gr33a¹/ +* ; *orco¹/ + ;* double heterozygous larvae on uncontaminated (N.C.) or *Ecc15*-contaminated yeast (OD₆₀₀ = 40). Statistical analyses were performed using a mixed-effects model (REML) including time and condition as fixed factors. Statistical comparisons between genotypes under *Ecc15*-contaminated conditions are shown (ns = not significant).
(PDF)

S15 Fig Larval exposure to *Ecc15*-contaminated food impairs development. Representative images showing adult emergence seven days after second-instar larvae were exposed to either uncontaminated yeast (N.C., top), yeast contaminated with *E. coli* (middle), or yeast contaminated with *Ecc15* (bottom), each applied at identical bacterial densities (OD_600_ = 40). Boxed regions are shown at higher magnification on the right. Images were acquired using a standard webcam.
(PDF)

S16 Fig Larval relocation behavior following exposure to *Ecc15*-contaminated food. (A) Schematic overview of the assay used to monitor larval movement from an *Ecc15*–yeast mixture toward an uncontaminated yeast source. (B–C) Quantification of the proportion of larvae located on yeast contaminated with increasing concentrations of *Ecc15* (OD_600_ = 5, 10, 20, and 40) (Food A), in the surrounding agar region (Out), or on the uncontaminated yeast (Food B). Measurements were taken at 0 h and 24 h (B) or at 0 h and 2 h (C) after transfer onto the initial food source. Both panels include a control condition in which Food A and Food B consisted of uncontaminated yeast, providing a baseline distribution in the absence of bacterial cues. Panel B additionally includes a condition in which both food patches were contaminated with *Ecc15* (OD_600_ = 40), testing larval distribution in the absence of an uncontaminated alternative food source. Data are presented as means ± SD. Individual points represent experimental replicates (each consisting of groups of ≥ 25 larvae). Statistical analyses were performed using Fisher’s exact test (ns = not significant; *p < 0.05; ***p < 0.001; ****p < 0.0001).
(PDF)

S17 Fig Baseline behavioral characterization of parental GAL4 and UAS lines on *Ecc15*-contaminated food. (A–F) Quantification over time of larval dispersal on *Ecc15*-contaminated yeast for parental and control lines used throughout the study. (A) *+>UAS-Shi*^*ts*^ and *orco-GAL4>+* larvae. (B) *+>UAS-Shi*^*ts*^ and *GH146-GAL4>+* larvae. (C) *+>UAS-Kir2.1* and *Gr66a-GAL4>+* larvae. (D) *+>UAS-Kir2.1* and *Gr33a-GAL4>+* larvae. (E) *+>UAS-Kir2.1* and *Or49a-GAL4>+* larvae. (F) Wild-type, *Gr33a¹; Gr33a-GAL4*, and *Gr33a¹; UAS-Gr33a* larvae. Statistical analyses were performed using a mixed-effects model (REML) with time and condition as fixed factors (ns = not significant; ****p < 0.0001). Pairwise comparisons between indicated conditions are shown.
(PDF)

S1 Data Raw data and statistical analyses for the main figures. Data are organized by figure and panel in separate sheets, following the labeling used in the article, to facilitate interpretation, reproduction, and reuse. Available on Figshare: https://figshare.com/s/dae0cac9818bf88e6371.
(XLSX)

S2 Data Raw data and statistical analyses for the supporting figures. Data are organized by figure and panel in separate sheets, following the labeling used in the article, to facilitate interpretation, reproduction, and reuse. Available on Figshare: https://figshare.com/s/9a6eb6fb6c183bd7f3f4.
(XLSX)

S1 Movie Larval behavior on uncontaminated yeast. Second-instar *Drosophila melanogaster* larvae (*w¹¹¹*^*8*^) were placed directly onto an uncontaminated yeast substrate. The video shows their behavior over the 3 h 30 min recording period. Images were acquired by time-lapse imaging (1 frame every 2 s) and compiled at 24 fps. Available on Figshare: https://figshare.com/s/54cf4674f0c1365e5b07.
(AVI)

S2 Movie Larval behavior on *Ecc15*-contaminated yeast. Second-instar larvae (*w¹¹¹*^*8*^) were placed directly onto yeast inoculated with *Ecc15* (OD_600_ = 40). The video shows their behavior over the 3 h 30 min recording period. Images were acquired by time-lapse imaging (1 frame every 2 s) and compiled at 24 fps. Available on Figshare: https://figshare.com/s/b143b90527a620300203.
(AVI)

S3 Movie Larval behavior on *E. coli*-contaminated yeast. Second-instar larvae *(w¹¹¹*^*8*^) were placed directly onto yeast inoculated with *E. coli* (OD_600_ = 40). The video shows their behavior over the 3 h 30 min recording period. Images were acquired by time-lapse imaging (1 frame every 2 s) and compiled at 24 fps. Available on Figshare: https://figshare.com/s/ebfbee471d060462bf31.
(AVI)
