# Supplementary material for: Multisensory pathogen detection drives rapid escape and shapes host–microbe interactions in Drosophila larvae
Source: PLoS Pathog. 2026 Aug 12;22(8):e1014506. doi: 10.1371/journal.ppat.1014506 (PMC13489533; doi:10.1371/journal.ppat.1014506)
Supplement: Supplementary file 1 [file ppat.1014506.s001.pdf]

A

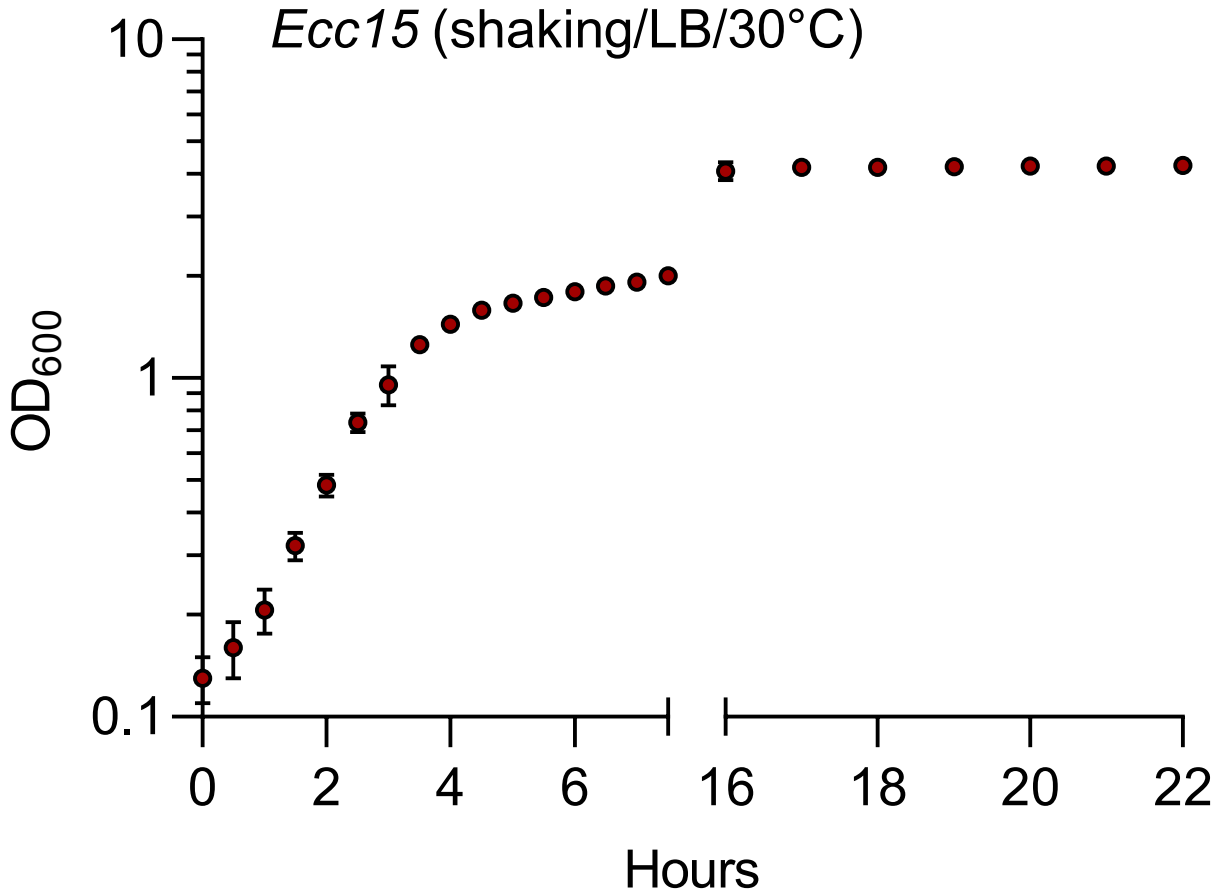

B

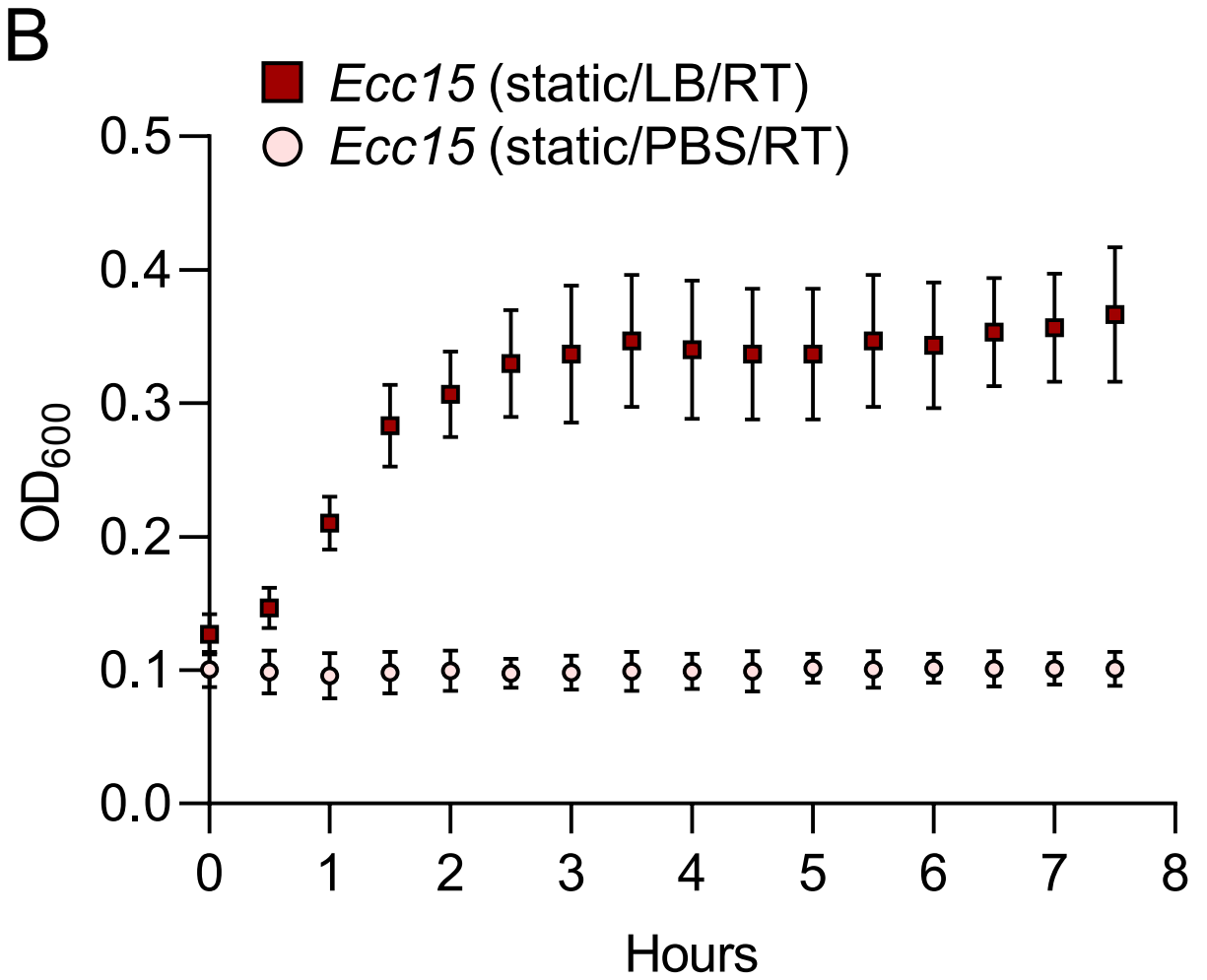

C

OD_{600}	Average *Ecc15* CFU/ml	SD
5	6,48E+08	1,08E+08
10	1,25E+09	1,03E+08
20	2,29E+09	1,35E+08
40	5,09E+09	1,05E+08

D

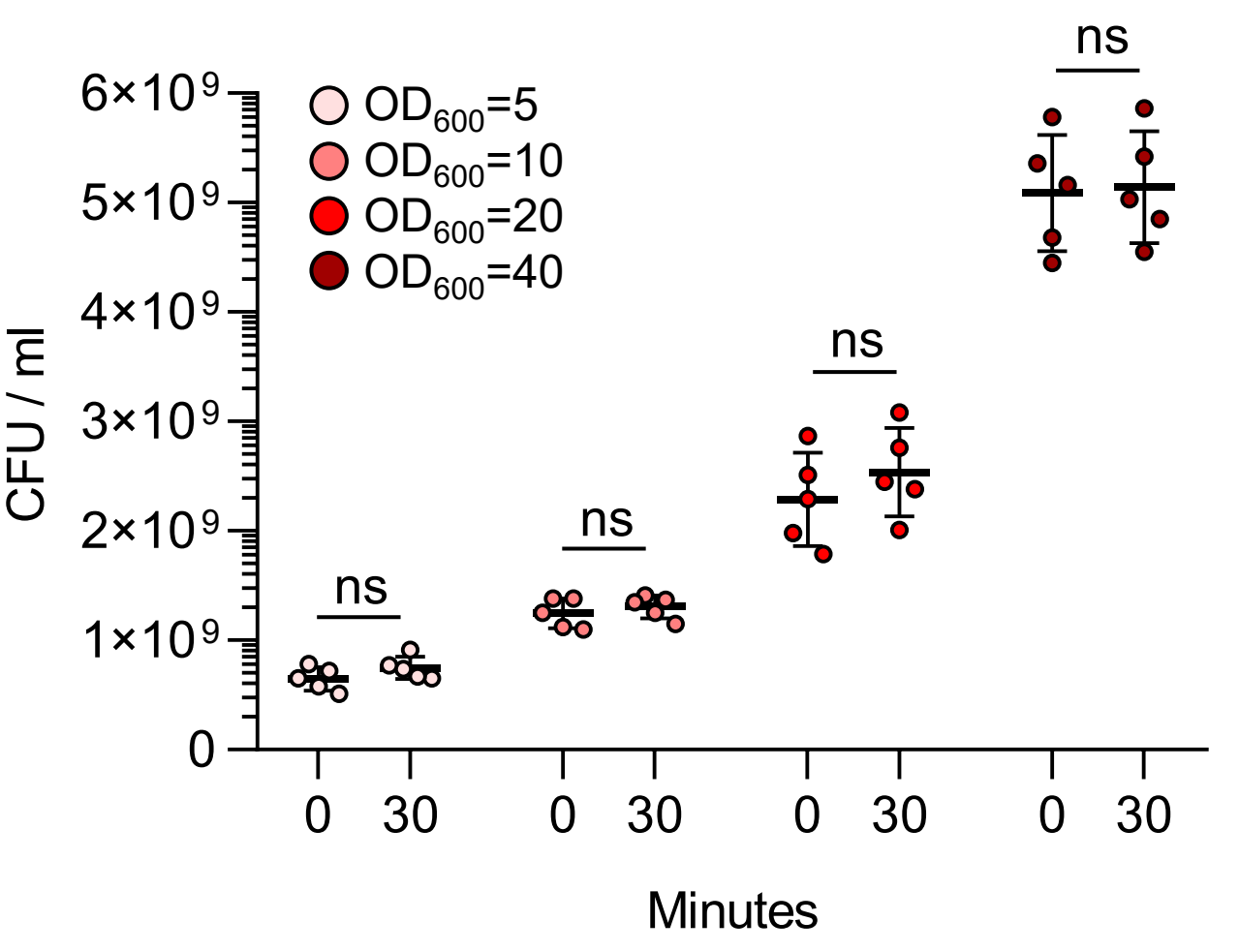

S1 Fig
