# Supplementary material for: Multisensory pathogen detection drives rapid escape and shapes host–microbe interactions in Drosophila larvae
Source: PLoS Pathog. 2026 Aug 12;22(8):e1014506. doi: 10.1371/journal.ppat.1014506 (PMC13489533; doi:10.1371/journal.ppat.1014506)
Supplement: Supplementary file 2 [file ppat.1014506.s002.pdf]

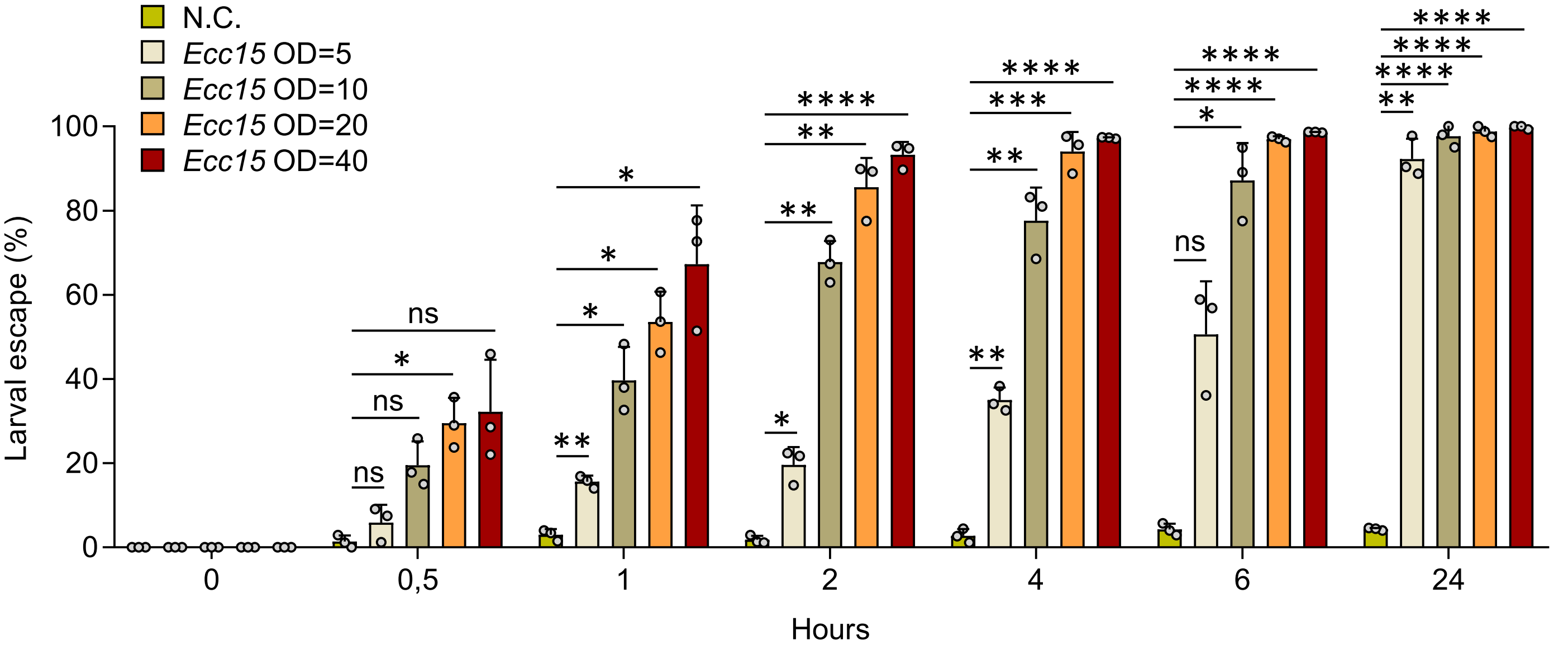
N.C.
Ecc15 OD=5
Ecc15 OD=10
Ecc15 OD=20
Ecc15 OD=40
Larval escape (%)
0
20
40
60
80
100
0
0,5
1
2
4
6
24
Hours
ns
*
**
***
****

S2 Fig
