# Supplementary material for: Multisensory pathogen detection drives rapid escape and shapes host–microbe interactions in Drosophila larvae
Source: PLoS Pathog. 2026 Aug 12;22(8):e1014506. doi: 10.1371/journal.ppat.1014506 (PMC13489533; doi:10.1371/journal.ppat.1014506)
Supplement: Supplementary file 3 [file ppat.1014506.s003.pdf]

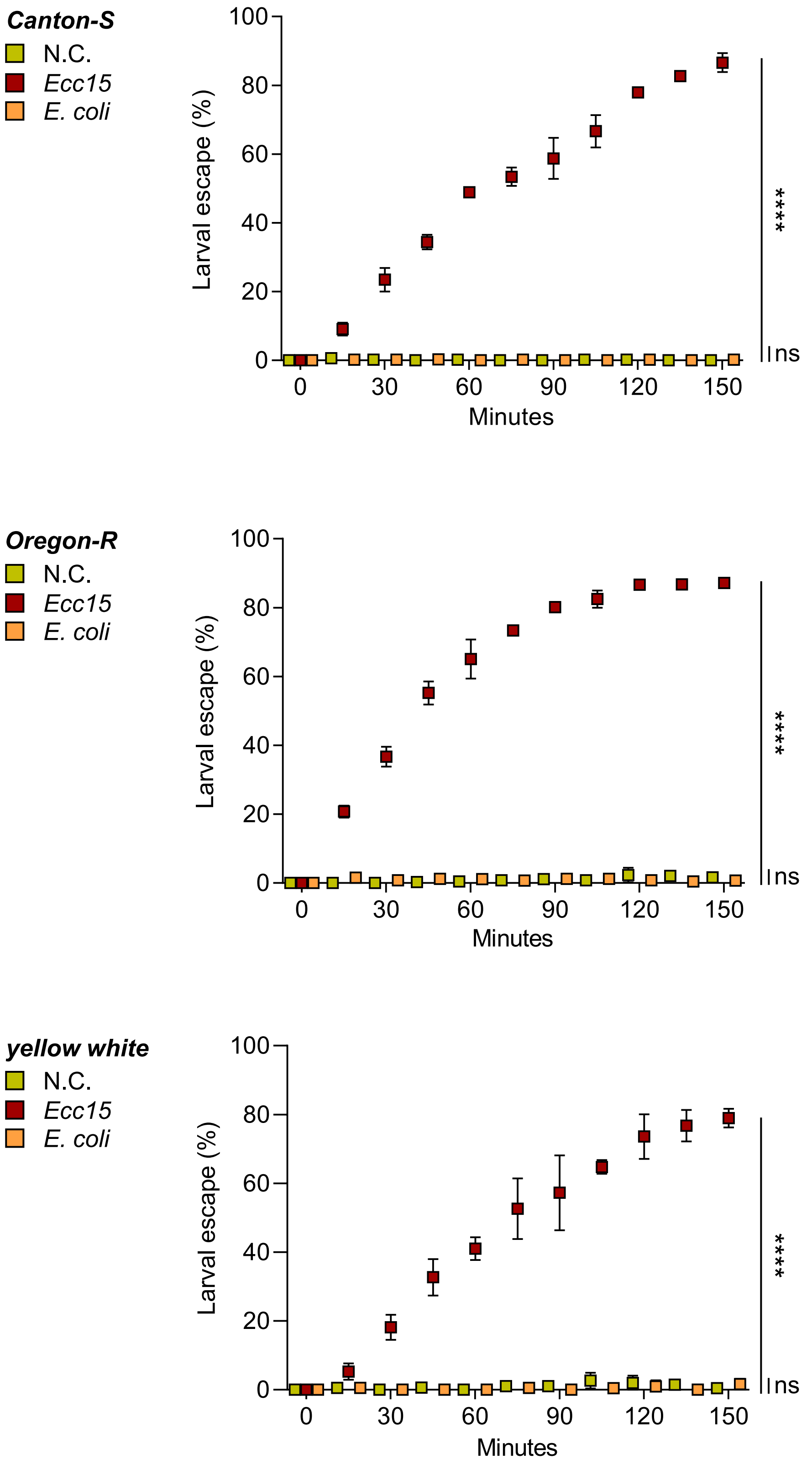
Canton-S
N.C.
Ecc15
E. coli
Larval escape (%)
Minutes
****
ns
Oregon-R
N.C.
Ecc15
E. coli
Larval escape (%)
Minutes
****
ns
yellow white
N.C.
Ecc15
E. coli
Larval escape (%)
Minutes
****
ns

S3 Fig
