# Supplementary material for: Multisensory pathogen detection drives rapid escape and shapes host–microbe interactions in Drosophila larvae
Source: PLoS Pathog. 2026 Aug 12;22(8):e1014506. doi: 10.1371/journal.ppat.1014506 (PMC13489533; doi:10.1371/journal.ppat.1014506)
Supplement: Supplementary file 4 [file ppat.1014506.s004.pdf]

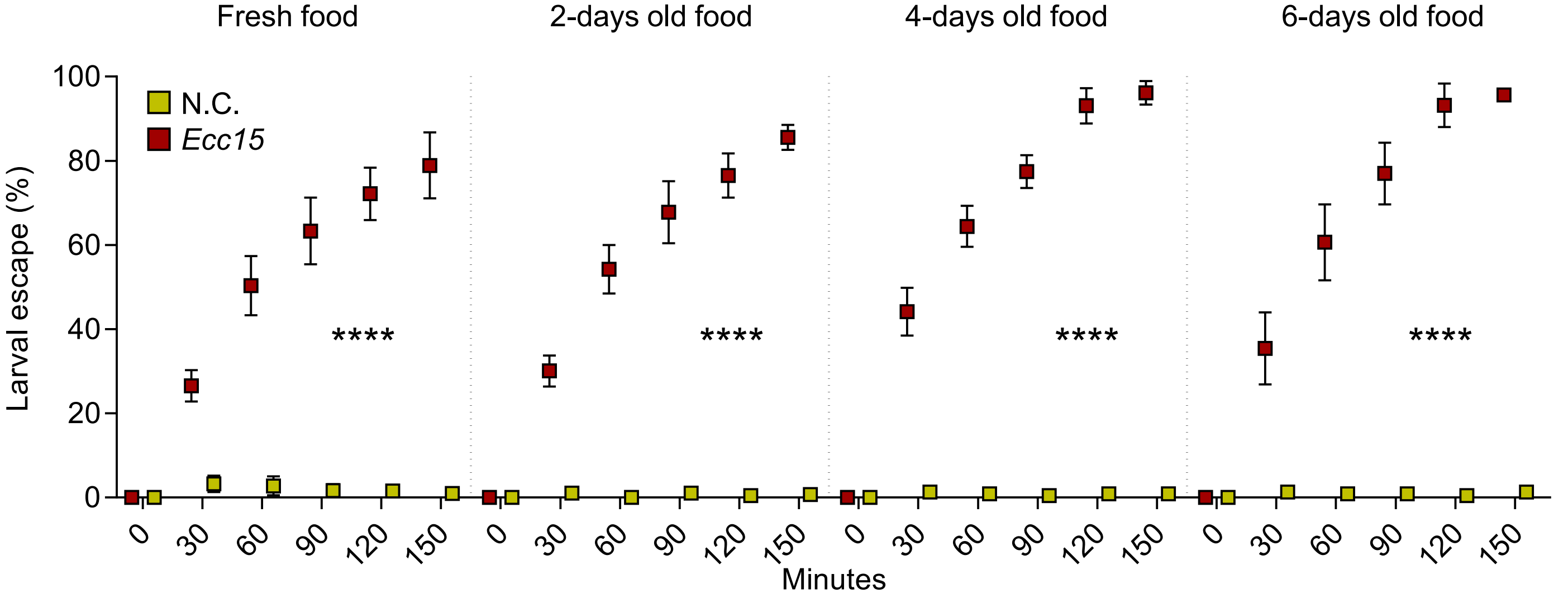
Fresh food
2-days old food
4-days old food
6-days old food
N.C.
Ecc15
Larval escape (%)
100
80
60
40
20
0
****
****
****
****
0
30
60
90
120
150
Minutes

S4 Fig
