# Supplementary material for: Multisensory pathogen detection drives rapid escape and shapes host–microbe interactions in Drosophila larvae
Source: PLoS Pathog. 2026 Aug 12;22(8):e1014506. doi: 10.1371/journal.ppat.1014506 (PMC13489533; doi:10.1371/journal.ppat.1014506)
Supplement: Supplementary file 5 [file ppat.1014506.s005.pdf]

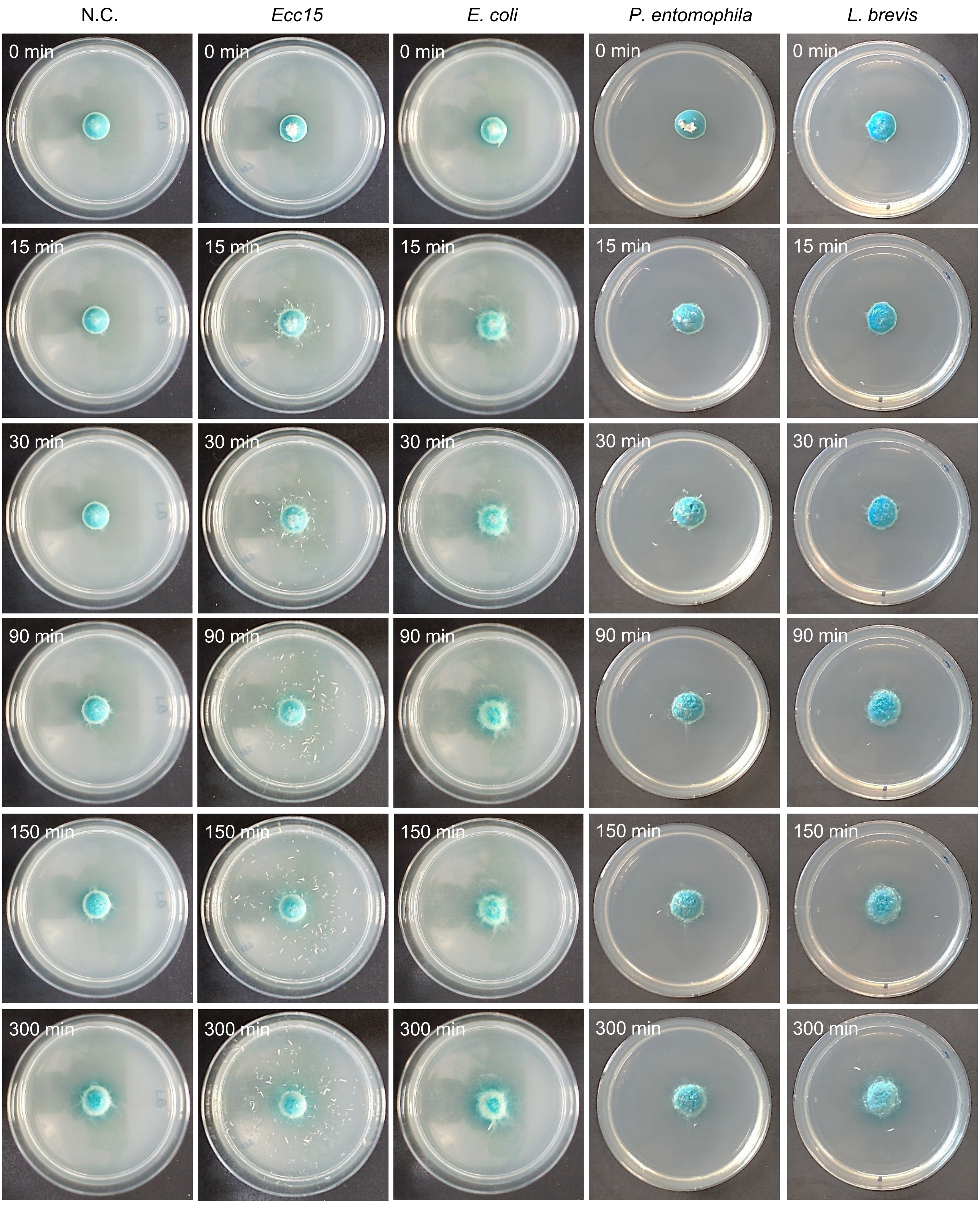
N.C.
Ecc15
E. coli
P. entomophila
L. brevis
0 min
15 min
30 min
90 min
150 min
300 min

S5 Fig
