# Supplementary material for: Multisensory pathogen detection drives rapid escape and shapes host–microbe interactions in Drosophila larvae
Source: PLoS Pathog. 2026 Aug 12;22(8):e1014506. doi: 10.1371/journal.ppat.1014506 (PMC13489533; doi:10.1371/journal.ppat.1014506)
Supplement: Supplementary file 6 [file ppat.1014506.s006.pdf]

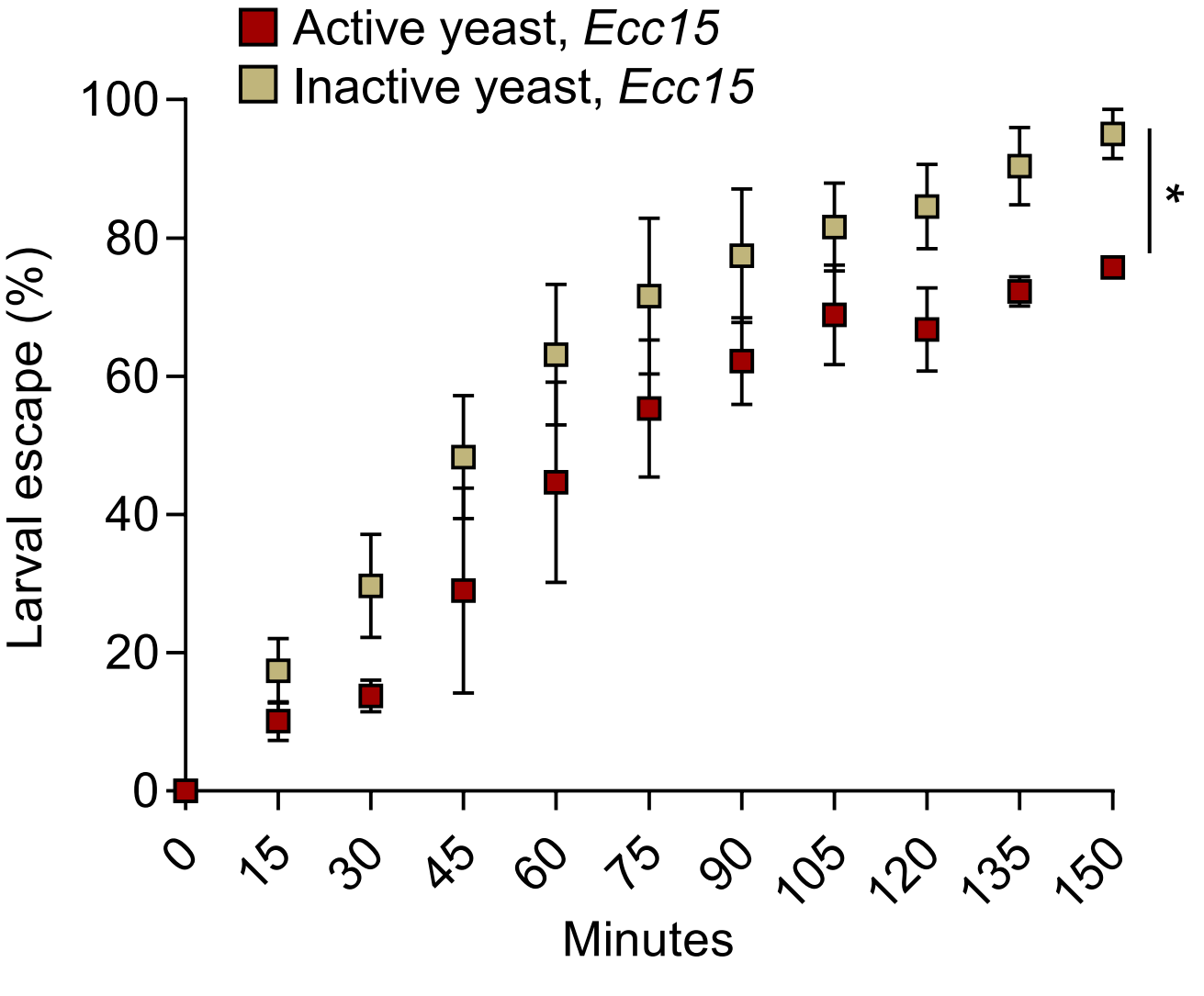

Active yeast, *Ecc15*
Inactive yeast, *Ecc15*
Larval escape (%)
100
80
60
40
20
0
*
0
15
30
45
60
75
90
105
120
135
150
Minutes

S6 Fig
