# Supplementary material for: Multisensory pathogen detection drives rapid escape and shapes host–microbe interactions in Drosophila larvae
Source: PLoS Pathog. 2026 Aug 12;22(8):e1014506. doi: 10.1371/journal.ppat.1014506 (PMC13489533; doi:10.1371/journal.ppat.1014506)
Supplement: Supplementary file 7 [file ppat.1014506.s007.pdf]

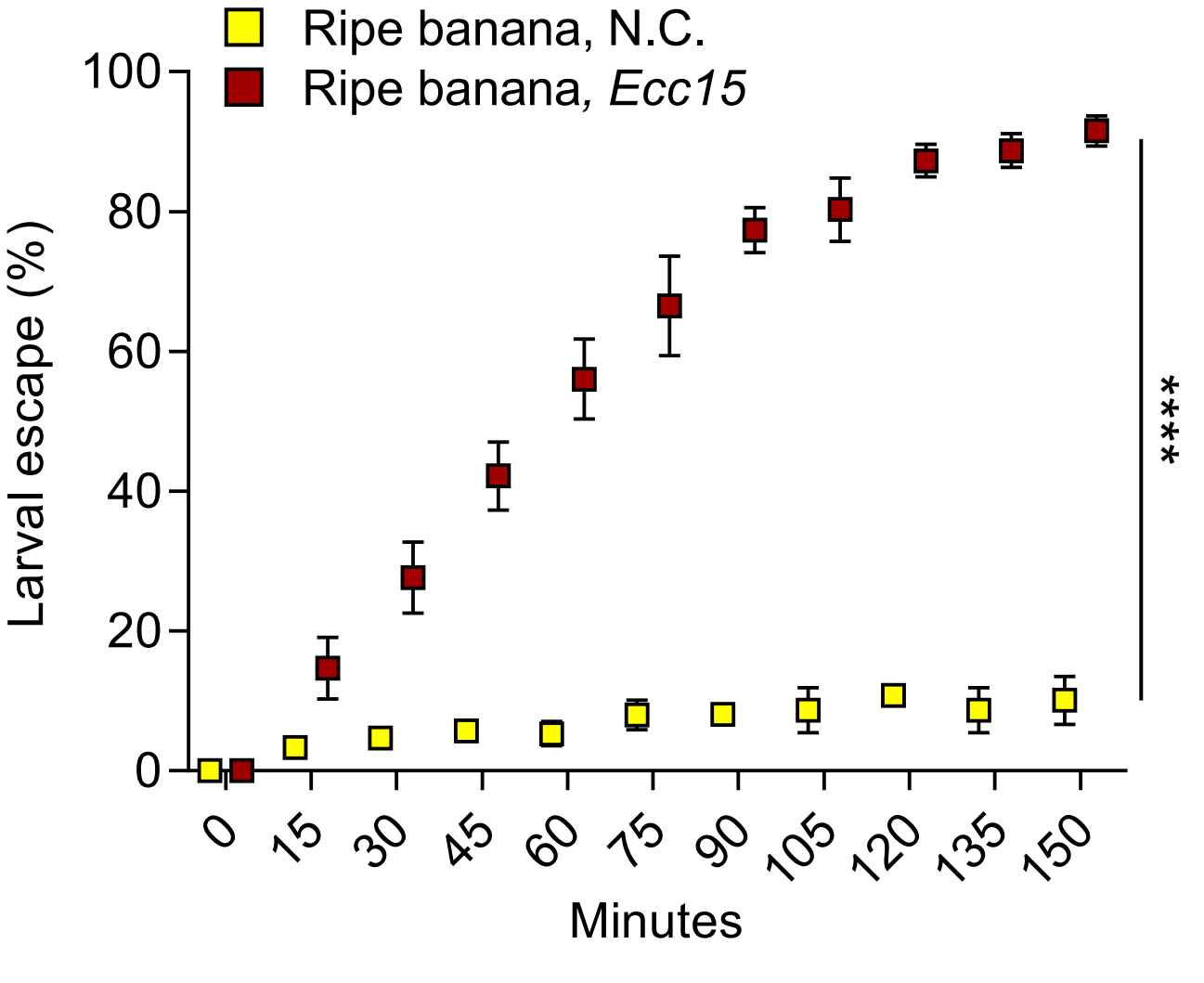
Ripe banana, N.C.
Ripe banana, *Ecc15*
100
80
60
40
20
0
Larval escape (%)
0
15
30
45
60
75
90
105
120
135
150
Minutes
****

S7 Fig
