# Supplementary material for: Multisensory pathogen detection drives rapid escape and shapes host–microbe interactions in Drosophila larvae
Source: PLoS Pathog. 2026 Aug 12;22(8):e1014506. doi: 10.1371/journal.ppat.1014506 (PMC13489533; doi:10.1371/journal.ppat.1014506)
Supplement: Supplementary file 8 [file ppat.1014506.s008.pdf]

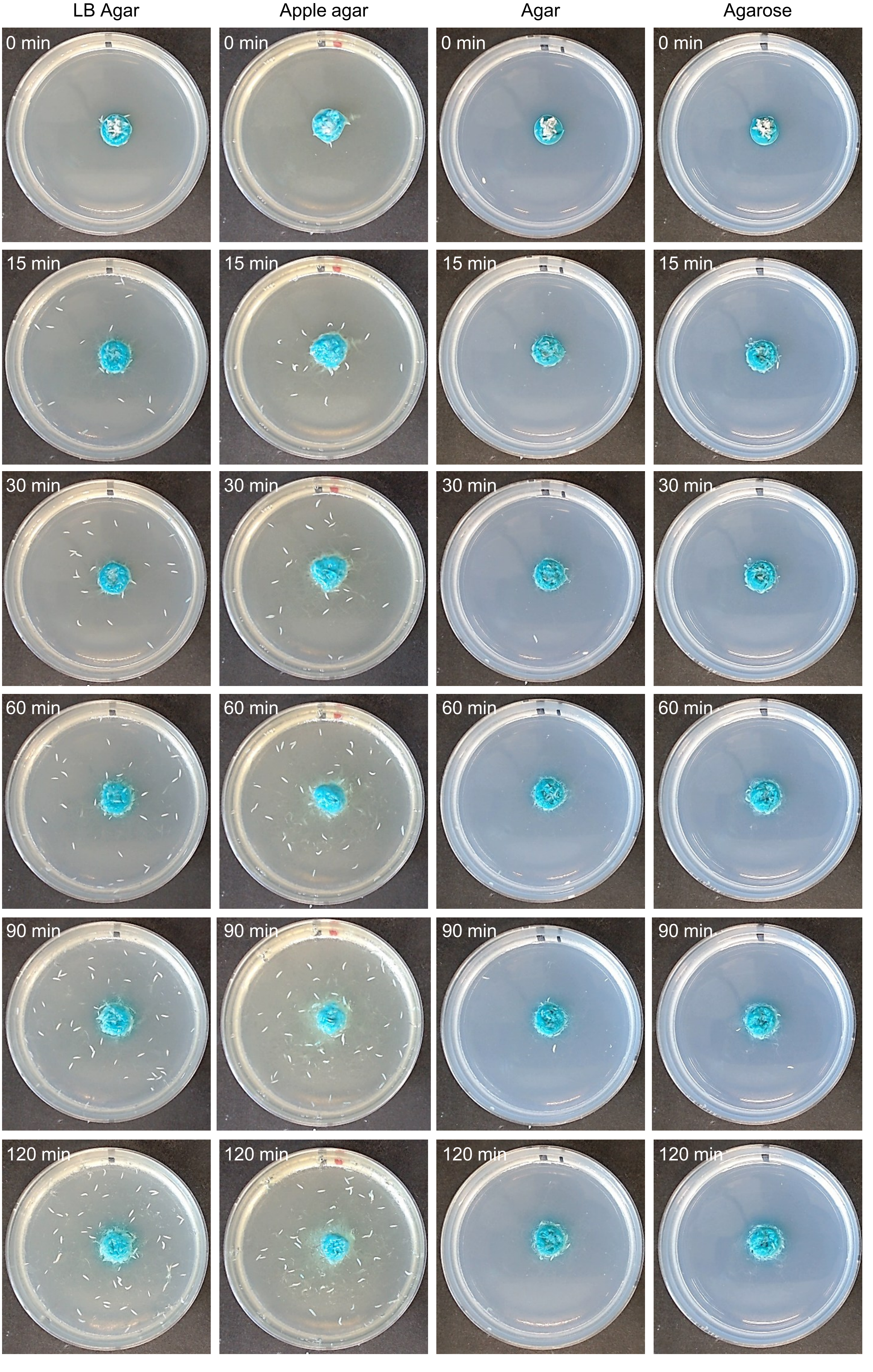
LB Agar
Apple agar
Agar
Agarose
0 min
15 min
30 min
60 min
90 min
120 min

S8 Fig
