# Supplementary material for: Multisensory pathogen detection drives rapid escape and shapes host–microbe interactions in Drosophila larvae
Source: PLoS Pathog. 2026 Aug 12;22(8):e1014506. doi: 10.1371/journal.ppat.1014506 (PMC13489533; doi:10.1371/journal.ppat.1014506)
Supplement: Supplementary file 9 [file ppat.1014506.s009.pdf]

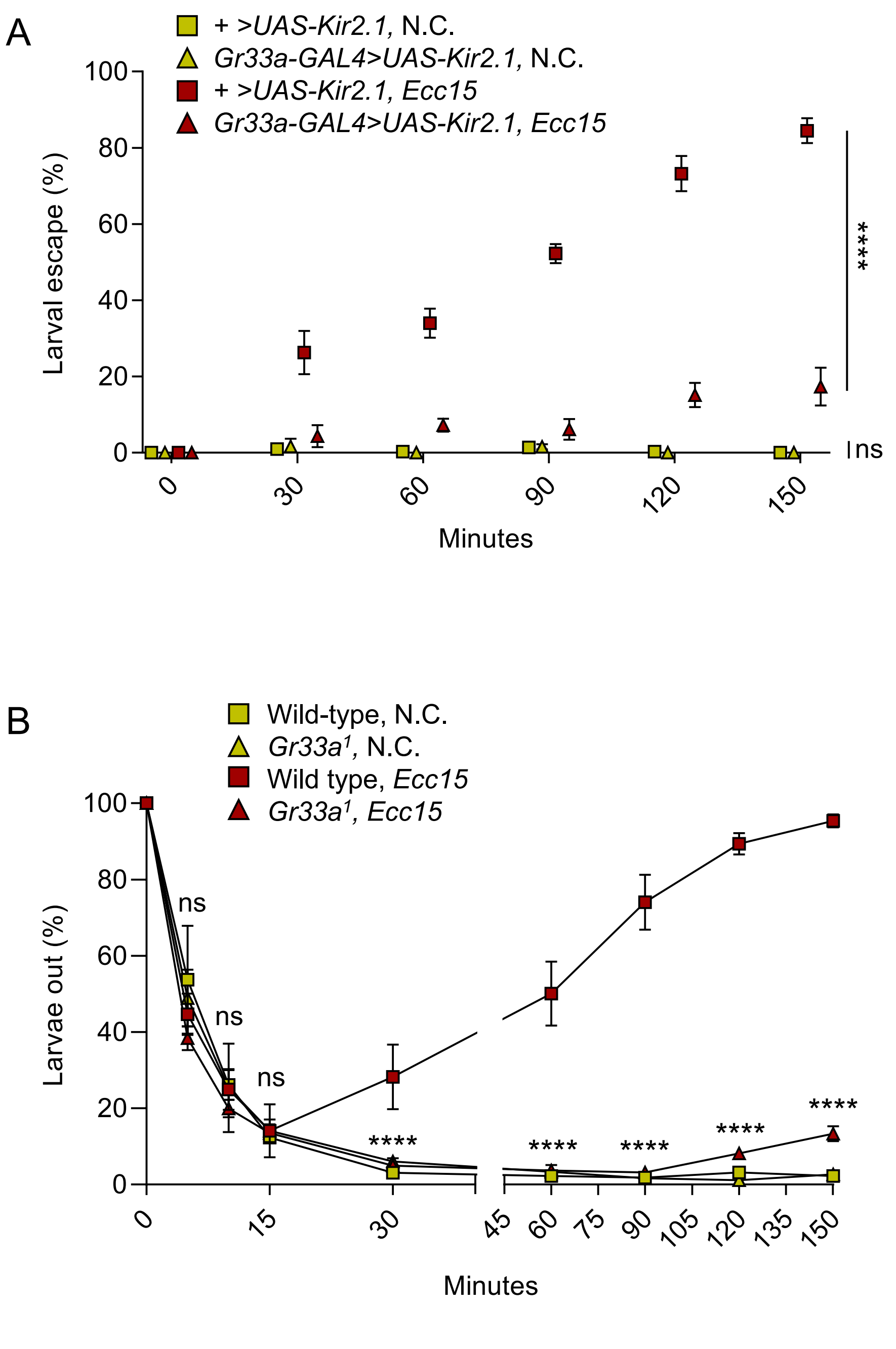
A
+ >UAS-Kir2.1, N.C.
Gr33a-GAL4>UAS-Kir2.1, N.C.
+ >UAS-Kir2.1, Ecc15
Gr33a-GAL4>UAS-Kir2.1, Ecc15
Larval escape (%)
Minutes
****
ns
B
Wild-type, N.C.
Gr33a1, N.C.
Wild type, Ecc15
Gr33a1, Ecc15
Larvae out (%)
Minutes
ns
ns
ns
****
****
****
****
****

S9 Fig
