# Supplementary material for: Multisensory pathogen detection drives rapid escape and shapes host–microbe interactions in Drosophila larvae
Source: PLoS Pathog. 2026 Aug 12;22(8):e1014506. doi: 10.1371/journal.ppat.1014506 (PMC13489533; doi:10.1371/journal.ppat.1014506)
Supplement: Supplementary file 10 [file ppat.1014506.s010.pdf]

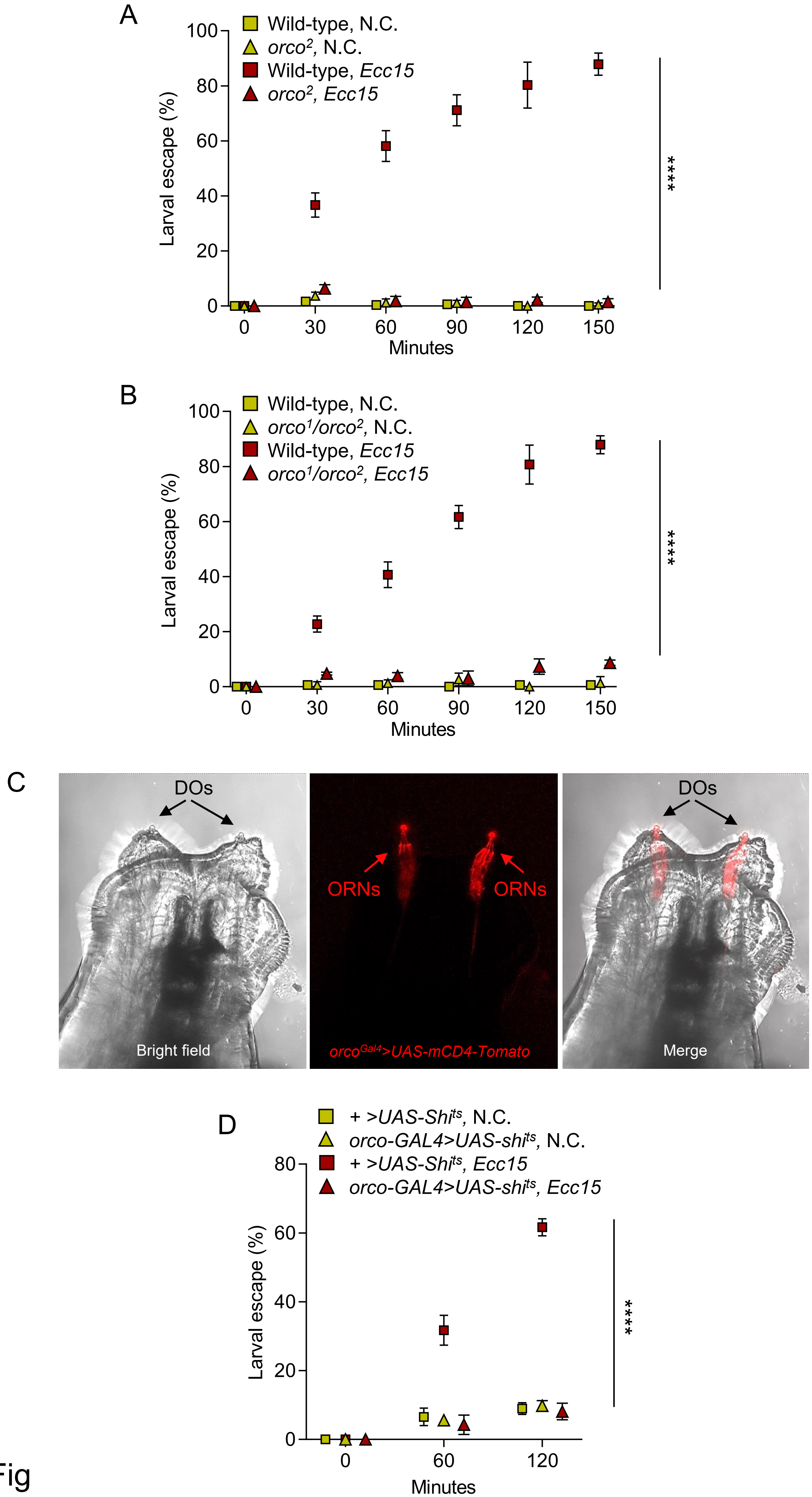

A
Wild-type, N.C.
orco2, N.C.
Wild-type, Ecc15
orco2, Ecc15
Larval escape (%)
Minutes
****
B
Wild-type, N.C.
orco1/orco2, N.C.
Wild-type, Ecc15
orco1/orco2, Ecc15
Larval escape (%)
Minutes
****
C
DOs
ORNs
Bright field
orcoGal4>UAS-mCD4-Tomato
Merge
D
+ >UAS-Shits, N.C.
orco-GAL4>UAS-shits, N.C.
+ >UAS-Shits, Ecc15
orco-GAL4>UAS-shits, Ecc15
Larval escape (%)
Minutes
****

S10 Fig
