# Supplementary material for: Multisensory pathogen detection drives rapid escape and shapes host–microbe interactions in Drosophila larvae
Source: PLoS Pathog. 2026 Aug 12;22(8):e1014506. doi: 10.1371/journal.ppat.1014506 (PMC13489533; doi:10.1371/journal.ppat.1014506)
Supplement: Supplementary file 11 [file ppat.1014506.s011.pdf]

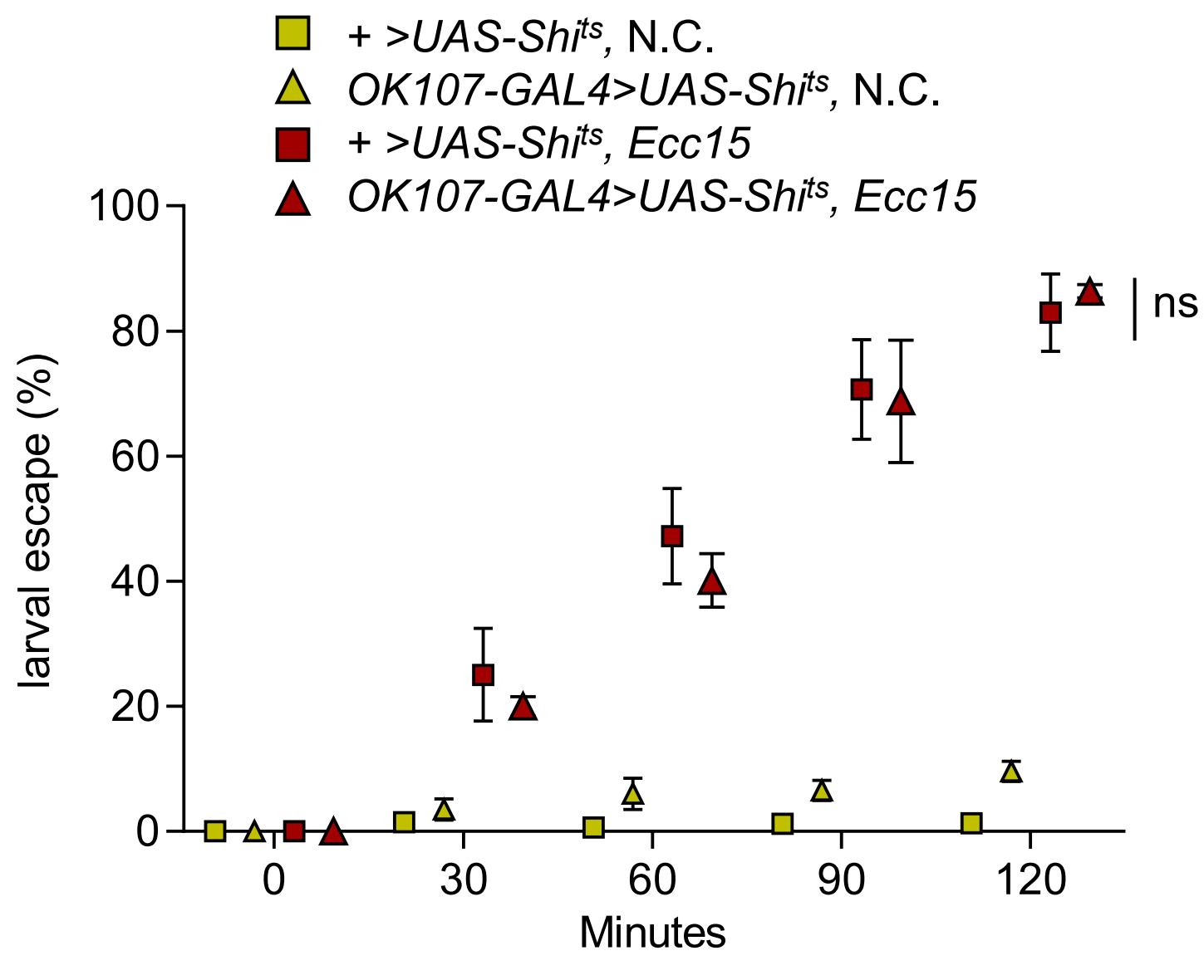

+ >UAS-Shi^ts, N.C.
OK107-GAL4>UAS-Shi^ts, N.C.
+ >UAS-Shi^ts, Ecc15
OK107-GAL4>UAS-Shi^ts, Ecc15
100
80
60
40
20
0
larval escape (%)
0
30
60
90
120
Minutes
ns

S11 Fig
