# Supplementary material for: Multisensory pathogen detection drives rapid escape and shapes host–microbe interactions in Drosophila larvae
Source: PLoS Pathog. 2026 Aug 12;22(8):e1014506. doi: 10.1371/journal.ppat.1014506 (PMC13489533; doi:10.1371/journal.ppat.1014506)
Supplement: Supplementary file 12 [file ppat.1014506.s012.pdf]

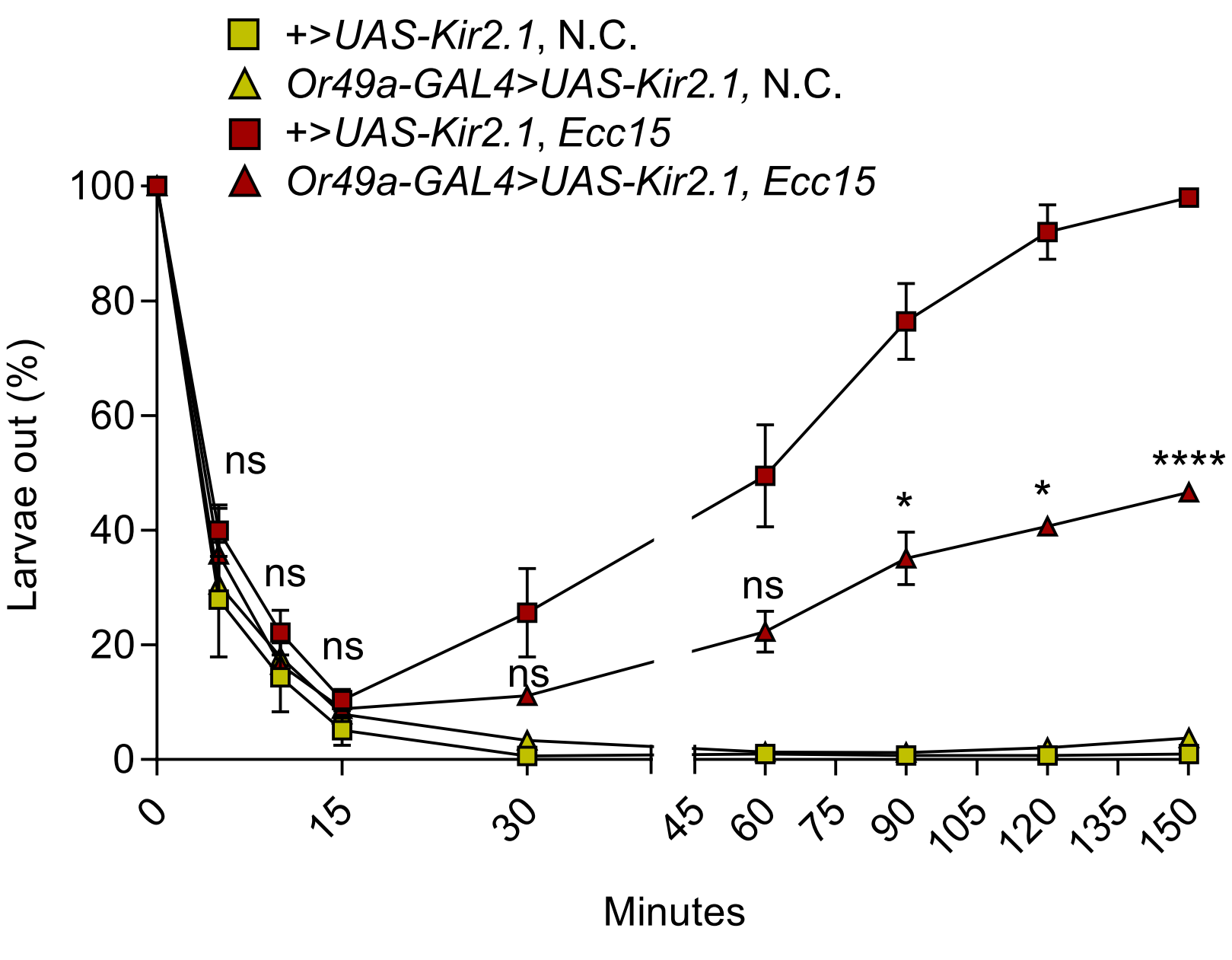
+>*UAS-Kir2.1*, N.C.
Or49a-GAL4>UAS-Kir2.1, N.C.
+>*UAS-Kir2.1*, *Ecc15*
Or49a-GAL4>UAS-Kir2.1, Ecc15
Larvae out (%)
100
80
60
40
20
0
ns
ns
ns
ns
ns
*
*
****
0
15
30
45
60
75
90
105
120
135
150
Minutes

S12 Fig
