# Supplementary material for: Multisensory pathogen detection drives rapid escape and shapes host–microbe interactions in Drosophila larvae
Source: PLoS Pathog. 2026 Aug 12;22(8):e1014506. doi: 10.1371/journal.ppat.1014506 (PMC13489533; doi:10.1371/journal.ppat.1014506)
Supplement: Supplementary file 13 [file ppat.1014506.s013.pdf]

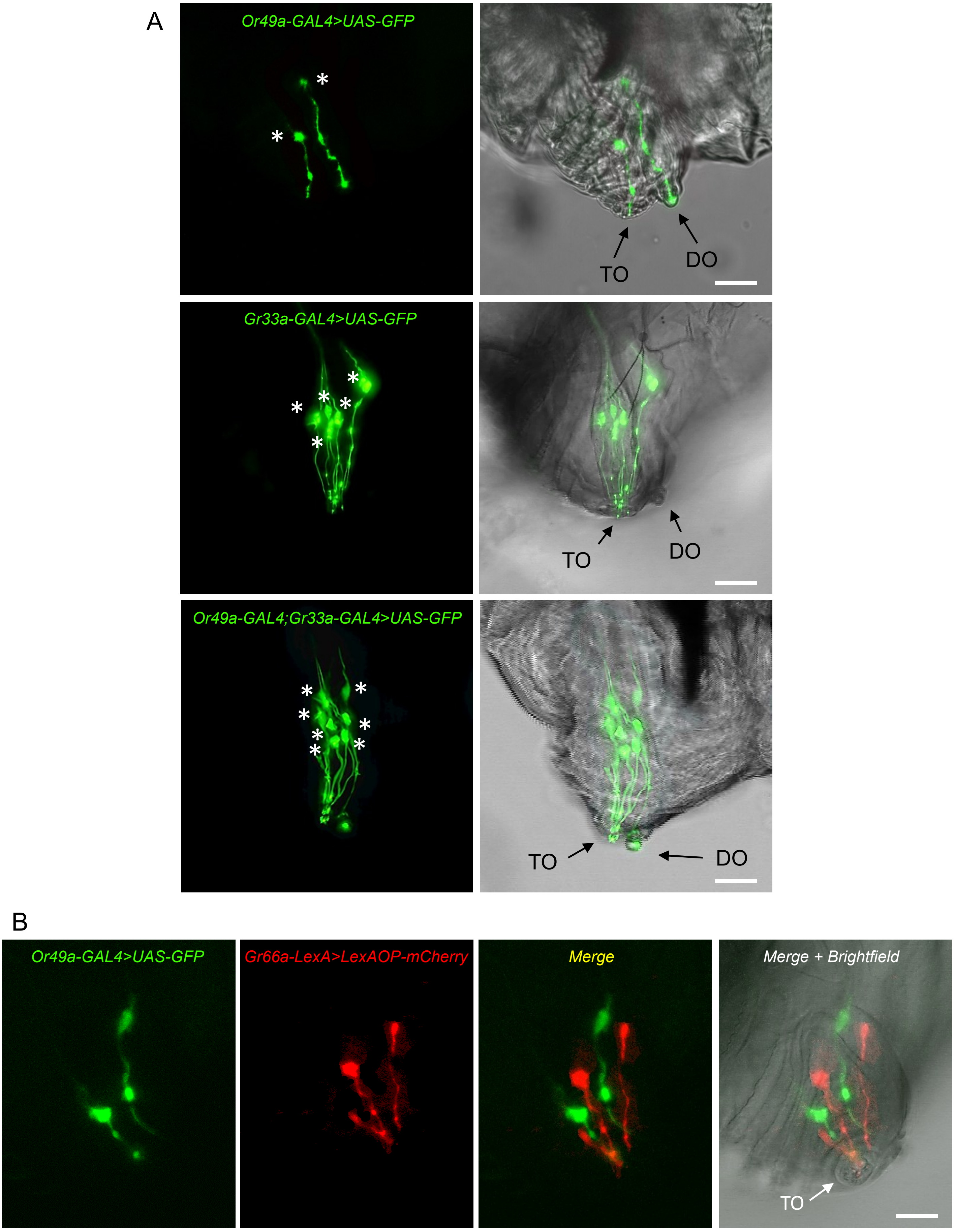

A
Or49a-GAL4>UAS-GFP
TO
DO
Gr33a-GAL4>UAS-GFP
TO
DO
Or49a-GAL4;Gr33a-GAL4>UAS-GFP
TO
DO
B
Or49a-GAL4>UAS-GFP
Gr66a-LexA>LexAOP-mCherry
Merge
Merge + Brightfield
TO

S13 Fig
