# Supplementary material for: Multisensory pathogen detection drives rapid escape and shapes host–microbe interactions in Drosophila larvae
Source: PLoS Pathog. 2026 Aug 12;22(8):e1014506. doi: 10.1371/journal.ppat.1014506 (PMC13489533; doi:10.1371/journal.ppat.1014506)
Supplement: Supplementary file 14 [file ppat.1014506.s014.pdf]

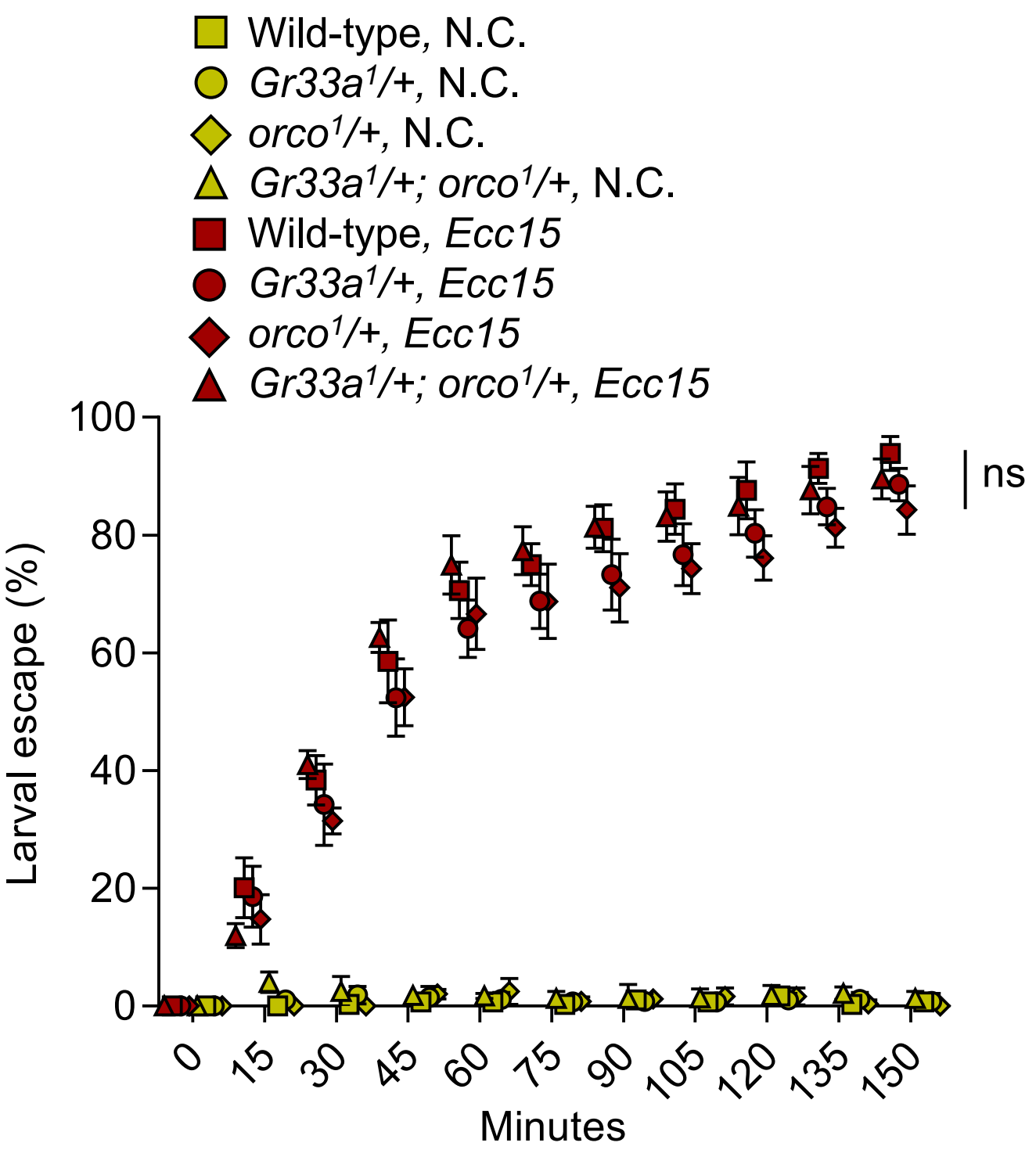
Wild-type, N.C.
Gr33a¹/+, N.C.
orco¹/+, N.C.
Gr33a¹/+; orco¹/+, N.C.
Wild-type, *Ecc15*
Gr33a¹/+, *Ecc15*
orco¹/+, *Ecc15*
Gr33a¹/+; orco¹/+, *Ecc15*
Larval escape (%)
100
80
60
40
20
0
ns
0
15
30
45
60
75
90
105
120
135
150
Minutes

S14 Fig
