# Supplementary material for: Multisensory pathogen detection drives rapid escape and shapes host–microbe interactions in Drosophila larvae
Source: PLoS Pathog. 2026 Aug 12;22(8):e1014506. doi: 10.1371/journal.ppat.1014506 (PMC13489533; doi:10.1371/journal.ppat.1014506)
Supplement: Supplementary file 16 [file ppat.1014506.s016.pdf]

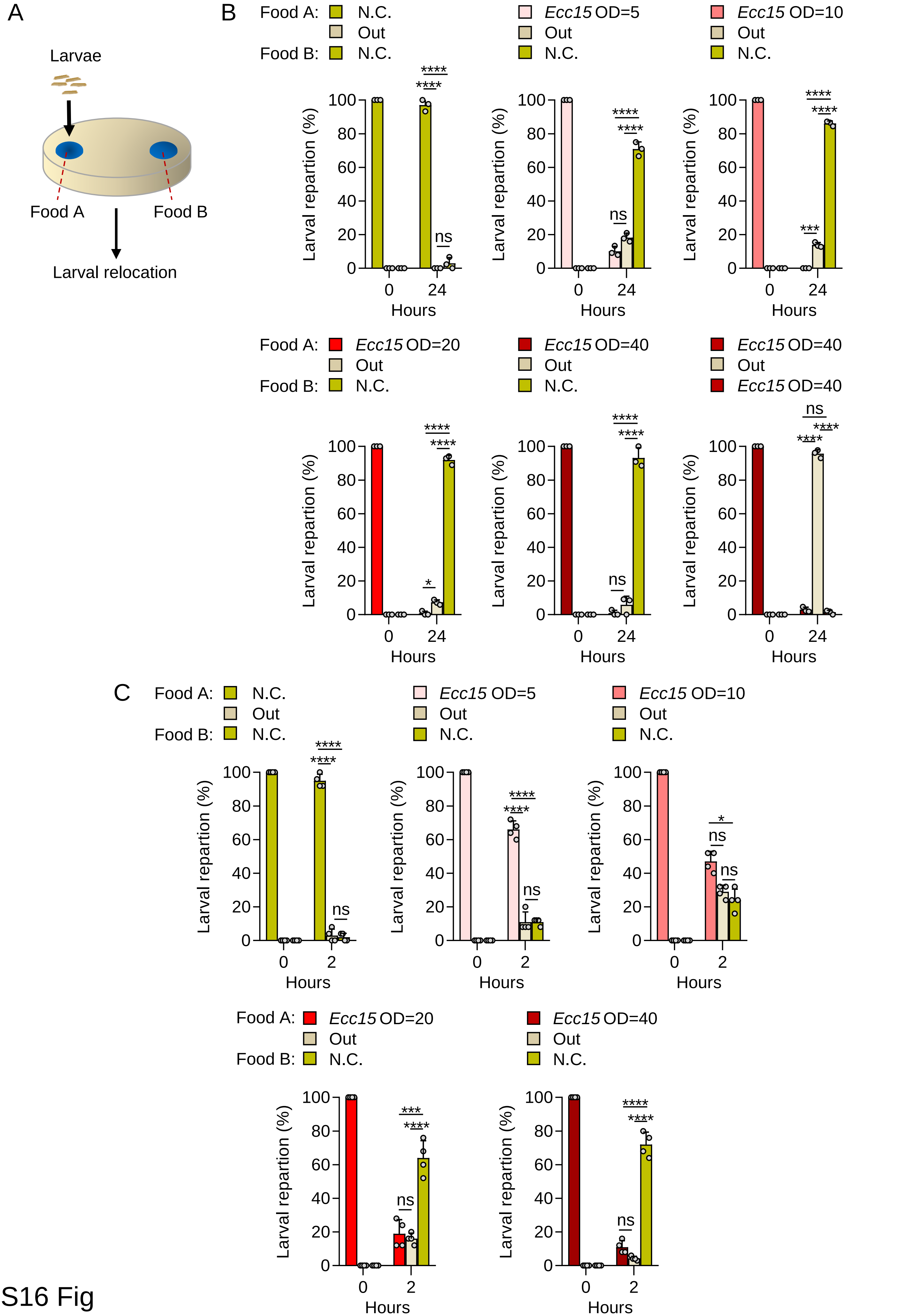
A
Larvae
Food A
Food B
Larval relocation
B
Food A: N.C. / Out / Food B: N.C.
Food A: Ecc15 OD=5 / Out / Food B: N.C.
Food A: Ecc15 OD=10 / Out / Food B: N.C.
Food A: Ecc15 OD=20 / Out / Food B: N.C.
Food A: Ecc15 OD=40 / Out / Food B: N.C.
Food A: Ecc15 OD=40 / Out / Food B: Ecc15 OD=40
Larval repartion (%)
Hours
C
Food A: N.C. / Out / Food B: N.C.
Food A: Ecc15 OD=5 / Out / Food B: N.C.
Food A: Ecc15 OD=10 / Out / Food B: N.C.
Food A: Ecc15 OD=20 / Out / Food B: N.C.
Food A: Ecc15 OD=40 / Out / Food B: N.C.
Larval repartion (%)
Hours

S16 Fig
