# Supplementary material for: Multisensory pathogen detection drives rapid escape and shapes host–microbe interactions in Drosophila larvae
Source: PLoS Pathog. 2026 Aug 12;22(8):e1014506. doi: 10.1371/journal.ppat.1014506 (PMC13489533; doi:10.1371/journal.ppat.1014506)
Supplement: Supplementary file 17 [file ppat.1014506.s017.pdf]

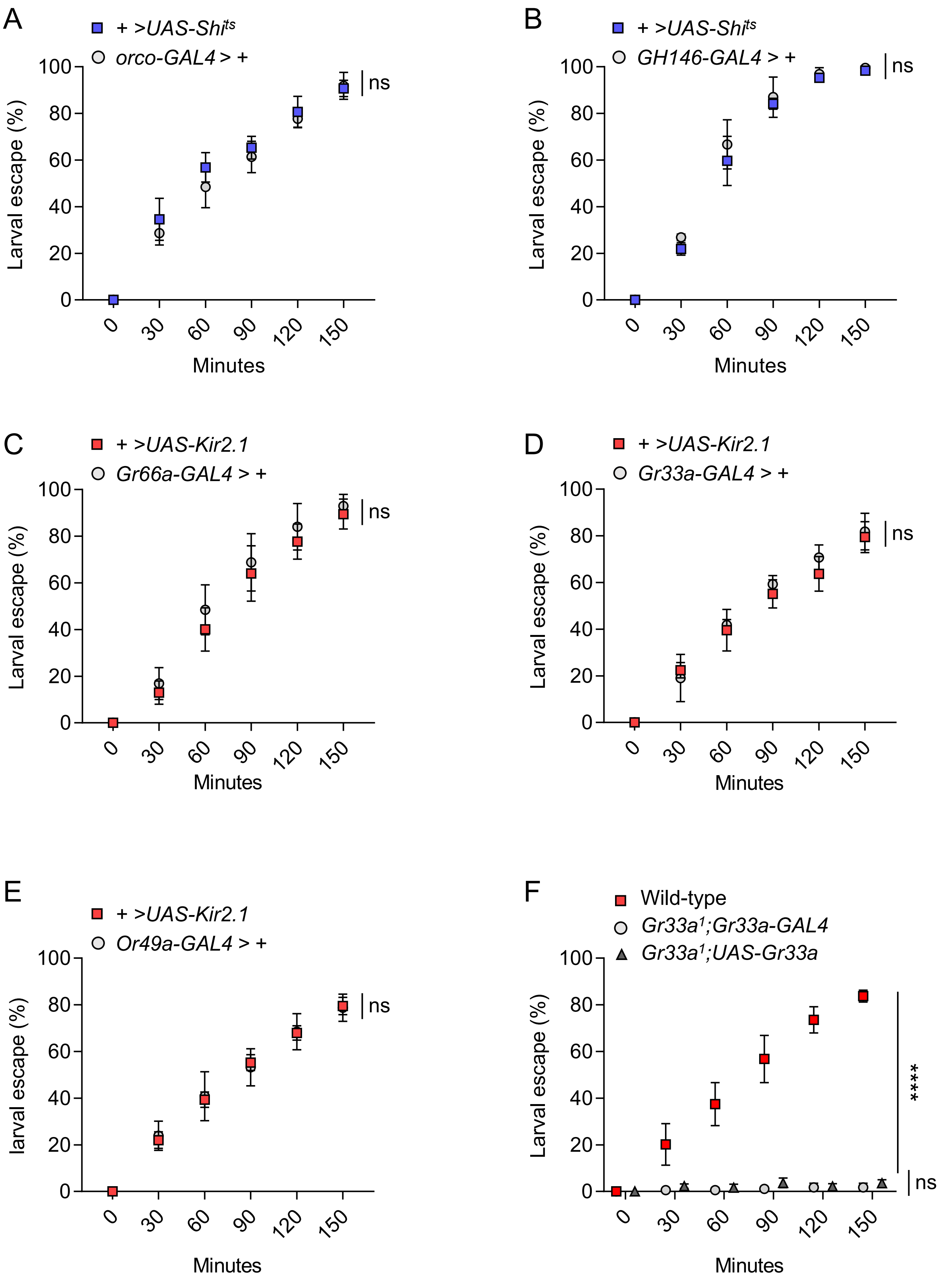
A
+ >UAS-Shi^{ts}
orco-GAL4 > +
ns
Larval escape (%)
Minutes
B
+ >UAS-Shi^{ts}
GH146-GAL4 > +
ns
Larval escape (%)
Minutes
C
+ >UAS-Kir2.1
Gr66a-GAL4 > +
ns
Larval escape (%)
Minutes
D
+ >UAS-Kir2.1
Gr33a-GAL4 > +
ns
Larval escape (%)
Minutes
E
+ >UAS-Kir2.1
Or49a-GAL4 > +
ns
larval escape (%)
Minutes
F
Wild-type
$Gr33a^{1}$;Gr33a-GAL4
$Gr33a^{1}$;UAS-Gr33a
****
ns
Larval escape (%)
Minutes
0
20
40
60
80
100
0
30
60
90
120
150

S17 Fig
